# The Detailed Balance Property and Chemical Systems out of Equilibrium

**DOI:** 10.1007/s11538-025-01487-1

**Published:** 2025-07-01

**Authors:** E. Franco, J. J. L. Velázquez

**Affiliations:** University of Bonn: Rheinische Friedrich-Wilhelms-Universitat Bonn, Bonn, Germany

**Keywords:** Chemical reaction networks, Detailed balance, Kinetic systems with fluxes, Non-equilibrium systems

## Abstract

The detailed balance property is a fundamental property that must be satisfied in all the macroscopic systems with a well defined temperature at each point. On the other hand, many biochemical networks work in non-equilibrium conditions and they can be effectively modelled using sets of equations in which the detailed balance condition fails. In this paper we study a class of "out of equilibrium" chemical networks that can be obtained freezing the concentration of some substances in chemical networks for which the detailed balance property holds. In particular, we prove that any chemical system with bidirectional chemical reactions can be extended to a system having additional substances and for which the detailed balance property holds.

## Introduction

A property that must be satisfied at the fundamental level by any chemical system with well defined thermodynamic quantities at each macroscopic point, as the temperature and the concentration of substances, is that the chemical rates should satisfy the so-called detailed balance property. This property, that was introduced for the analysis of collisions in gases by Boltzmann (see Boltzmann (1964)), states that at equilibrium each reaction is balanced by its reverse reaction. However, many biochemical networks modelling biological processes contain irreversible reactions and therefore they do not satisfy the detailed balance condition. One way of justifying these models is to assume that they describe an open system, which is in contact with one or more reservoirs of substances. As a consequence these biochemical networks operate in "out of equilibrium" conditions.

In this paper we will be concerned only with chemical systems in which the temperature is constant, therefore the systems that we study are assumed to be in contact with a reservoir at constant temperature. The chemical systems that we consider exchange heat with the environment. We restrict the attention to chemical networks that are endowed with the mass action kinetics with mass action kinetics, hence to chemical networks with reaction rates given by the mass action law. We stress that we make this choice partly because the mass action assumption simplifies the analysis of the kinetic systems and partly because, many non-mass action kinetics can be obtained as limits of mass action kinetics. This is the case for instance for the Michaelis-Menten kinetics or for the Hill law (see for instance (Goldbeter and Koshland 1981; Segel and Slemrod 1989)).

The kinetic systems that we consider are "out of equilibrium" due to the exchange of matter with the environment. In particular, we assume that these kinetic systems are in contact with substances whose concentration is out of equilibrium. Under some assumptions that will be prescribed later, these systems can be described by effective models in which the detailed balance property fails. From this point of view, the failure of detailed balance provides a measure for the lack of equilibrium of the system.

One of the issues that we address is how to derive kinetic systems that are out of equilibrium taking as a starting point kinetic systems that satisfy the detailed balance condition. In other words, we assume that the detailed balance property should be satisfied at the fundamental level by any chemical system and the lack of detailed balance takes place only as an effective property that arises from the fact that the concentrations of some substances in the network are kept at "non equilibrium" values in an active manner. The reason why these concentrations are kept out of equilibrium could be, for instance, an exchange of chemicals between the systems under consideration (for instance a subset of chemicals inside a cell) and the environment which is assumed to be out of equilibrium. Or alternatively the concentration of a substance could be kept at non-equilibrium concentrations by means of an active mechanism, for instance the production of ATP in mitochondria.

Let us mention that kinetic systems that exchange chemicals with the environment can be found in the mathematical and physical literature. We can refer for instance to Craciun and Feinberg (2005, 2006, 2010); Schnakenberg (1976). One of the questions that we examine in this paper is the following. Suppose that we have a kinetic system that satisfies the detailed balance property. Suppose that some concentrations are frozen at constant values. Then the non-frozen concentrations solve the equations associated with a kinetic system that we will denote as *reduced kinetic system*. As a matter of fact, it turns out that one of the ways in which it is possible to obtain a reduced kinetic system satisfying the detailed balance condition, is assuming that the frozen concentrations are at equilibrium values. Alternative ways to obtain a reduced kinetic system that satisfies the detailed balance property are also discussed in Section 5.2. In particular, we prove that the reduced system satisfies the detailed balance property in a robust way (i.e. the property is stable under small changes of the reaction rates and of the values of frozen concentrations) if and only if some topological condition on the reactions belonging to the cycles are satisfied both by the reduced and the original system. We remark that the fact that some topological conditions must be imposed on the cycles of the kinetic systems in order to obtain the detailed balance property in a robust manner is not surprising, as it is well known that the detailed balance property imposes conditions on the rates of the reactions that are part of a cycle (see for instance the *circuit condition* or *Wegscheider criterion* in Wegscheider (1902)).

As indicated above there is a plethora of models describing biochemical systems by means of ODEs for which the detailed balance condition is not satisfied. We can refer for instance to some models in Alon (2019) or the models of adaptation in Barkai and Leibler (1997); Ferrell (2016), the model of the Calvin cycle (see for instance (Gürbüz and Rendall 2022; Rendall and Velázquez 2014)), the kinetic proofreading model (see Franco and Velázquez (2025b); Hopfield (1974); Wong et al. (2018)), the model of ABC transporters (Flatt et al. 2023), the models of adaptation in Ferrell (2016) and some of the models in Milo and Phillips (2015) (for instance the models of chemotaxis) and in Phillips et al. (2012). Since we stated above that at the fundamental level every biochemical model should satisfy the detailed balance condition, it is natural to ask under which conditions a kinetic system can be obtained by means of the reduction of a kinetic system for which the detailed balance condition holds.

In this paper we will be mostly concerned with the study of systems in which all the reactions are bidirectional, i.e. they have the form

$$ (1) +(2) \leftrightarrows (3)+(4). $$

One-directional reactions have, instead the form

$$ (1) +(2) \rightarrow (3)+(4). $$

The models in which the detailed balance property holds are necessarily bidirectional. Conditions in one-directional networks that can be obtained as a limit of networks for which the detailed balance property holds have been found in Gorban and Yablonsky (2011).

We prove that any bidirectional biochemical system can be obtained as the reduction of a larger kinetic system for which the detailed balance property holds, we refer to this system as the *completed kinetic system*. More precisely, we prove that every open kinetic system can be obtained as the reduction of a *closed* kinetic system. A kinetic system is closed if it does not exchange substances with the environment. Since, as explained before, the lack of detailed balance emerges as an effective property of kinetic systems in which there are influxes and outfluxes of matter, closed kinetic systems are kinetic system that satisfy the detailed balance property and that do not exchange substances with the environment. The type of completion studied here has some similarities with the completion of a model of the Calvin cycle explained in Rendall (2017). In that paper, indeed, the relation between a model of Calvin cycle where the concentration of ATP molecules is assumed to change in time due to the reactions taking place in the network and a model in which the concentration of ATP molecules is chosen at constant values is studied. The concentration of ATP is assumed to be at constant values because the concentration of ATP is larger than the concentration of the other substances in the Calvin cycle. Here we focus on understanding whether or not the property of detailed balance of the completed system is inherited by the reduced system.

On the other hand, for some specific kinetic systems there might be constraints in the way in which reactions can be modified in order to obtain a closed completion, for instance because some chemical reactions are known in full detail. We prove that, when this is the case, it is not always possible to find a closed completion for these kinetic systems. Indeed, as we will explain later in detail, the fact that the kinetic system can be completed or not depends on the position of the reactions that cannot be modified in the network, in particular the difficulties arise when these reactions belong to cycles.

One of the main reasons to study the relation between the systems satisfying the detailed balance property and more general chemical networks is that this approach allows to measure the amount of "non-equilibrium" of these chemical networks by means of the fluxes of matter and energy required to make the system functioning. One of the issues in which we are interested is determining the degree of lack of equilibrium required for a chemical network to be able to perform some biological function. One example of this type of function is the so-called adaptation property (i.e. the capability of a network to react to changes in the signal, instead of reacting to the absolute value of it. This property is exhibited by many biological sensory systems ranging from bacteria to multicellular organisms). In Franco and Velázquez (2025a) we will prove that the adaptation property cannot take place for closed chemical systems in a robust manner. The relation between the adaptation property and the detailed balance property will be studied in detail in Franco and Velázquez (2025a).

**Plan of the paper and main results**

In Section 2 we will review some of the basic definitions and results on kinetic and chemical systems. In Subsection 2.1 we shall recall some of the basics concepts used in the theory of chemical networks, as for instance the notion of stoichiometric subspace, and of conservation laws. In Subsection 2.2 we will review the definition of kinetic systems (i.e. chemical networks associated with a rate function).

In Section 3 we review some of the main results for kinetic systems that satisfy the detailed balance property. The reason why we revise the properties of kinetic systems for which the detailed balance condition holds is that, as mentioned above, this property is a fundamental property of kinetic systems describing reactions in systems with well defined thermodynamic quantities (in local thermal equilibrium). In Subsection 3.1 and Subsection 3.2 we recall the definition of detailed balance and we recall that this property allows to associate to every substance $i \in \Omega$ in the kinetic system an energy $E_i$.

Finally, we recall that it is possible to associate to kinetic systems satisfying the detailed balance property a non-increasing free energy. Among the class of concentrations in the same stoichiometric class, the energy is minimized at the steady states. This means that when the kinetic system is at the steady state, then it does not dissipate energy. Moreover, the non-increasing free energy serves as a Lyapunov functional that allows to study the long-time behaviour of kinetic systems with the detailed balance property. Finally, we introduce the definition of closed kinetic systems.

In Section 4 we define the reduced kinetic systems and study some of their properties. We start by explaining how we define the reduction of the reactions of a chemical network. This is done in Subsection 4.1. In Subsection 4.2 we study the relation between the cycles of the reduced chemical network and the cycles of the non-reduced one. Notice that we are interested in the cycles of the kinetic systems because the detailed balance property is satisfied by a kinetic system if and only if the so-called circuit condition is satisfied along the cycles (Wegscheider 1902). As a consequence, if a chemical system does not have cycles and also its reduction does not have cycles, then the associated kinetic systems will satisfy the detailed balance property for every choice of reaction rates. In Subsection 4.3 we introduce the definition of reduced kinetic system, these are reduced chemical system endowed with reaction rates that depend on both the concentration of the frozen substances and the reaction rates of the non-reduced kinetic system.

In Section 5 we study under which conditions the reduction of a kinetic system that satisfies the detailed balance property also satisfies the detailed balance condition and under which conditions it does not. The main results in Subsection 5.1 are summarized in the following informal theorem that will be stated later precisely (Proposition 5.1).

### Theorem 1.1

Consider a kinetic system that satisfies the detailed balance property. Assume that the concentrations of some substances are kept at constant values by influxes and outfluxes of chemicals. Moreover, assume that if the substances whose concentrations are frozen appear in the cycles, then their concentrations are at equilibrium values. Then the corresponding reduced kinetic system satisfies the detailed balance property.

Here with equilibrium values we mean that the concentrations of the frozen substances are given by $$e^{- E} $$, where *E* is a vector of energies associated to the non-reduced kinetic system.

The main result of Subsection 5.2 is summarized in the following informal theorem. (This theorem summarizes the results in Theorem 5.4, Proposition 5.6, Proposition 5.8, Proposition 5.10).

### Theorem 1.2

Consider a kinetic system that satisfies the detailed balance property. Then the reduced kinetic system obtained by freezing some concentrations does not satisfy the detailed balance property unless either the reaction rates are fine tuned, or the frozen concentrations are chosen at equilibrium values, or the cycles of the reduced system and of the non reduced system are the same.

In Section 6 instead we investigate whether a kinetic system that is not closed admits a completion that is closed. The main result of this section is Theorem 6.3, which informally written states the following.

### Theorem 1.3

Consider a kinetic system that is not closed. Then it can be obtained as the reduction of a closed kinetic system.

This theorem guarantees the admissibility of any bidirectional chemical network. Here an admissible network is a network that is not closed, but can be obtained as the reduction of a closed kinetic system.

In Section 7 we define kinetic systems with fluxes. These are kinetic systems in which we have influxes and outfluxes of chemicals. Let us stress that in this paper we study kinetic systems with fluxes that keep the concentration of some substances constant in time. As a consequence, if we focus on the substances that change in time, the dynamics of kinetic systems with fluxes and of the reduced kinetic systems is the same and therefore kinetic systems with fluxes are just reformulations of reduced kinetic systems. The advantage of working with kinetic systems with fluxes instead of with reduced kinetic systems is that it is possible to obtain that the free energy of the kinetic system with fluxes *F* satisfies the following equality

$$ \partial _t F = -\mathcal {D}_R + J^{ext}. $$

Here $$\mathcal {D}_R $$ is the dissipation of free energy of the reduced kinetic system, while $$J^{ext} $$ is the contribution to the free energy due to the external fluxes.

### Notation

We define $\mathbb {R}_+$ and $\mathbb {R}_*$ to be given by $\mathbb {R}_* = [0, \infty )$ and $\mathbb {R}_+ = (0, \infty )$ respectively. In some cases, to help the reader we indicate with ${\textbf {0}}_d$ the zero vectors belonging to $\mathbb {R}^d$. Moreover we denote with $e_i \subset \mathbb {R}^n$, for $i \in 1, \dots n$, the vectors of the canonical basis of $\mathbb {R}^n$. Given two vectors $v_1, v_2 \in \mathbb {R}^n$ we denote with $\langle v_1, v_2 \rangle$ their euclidean scalar product in $\mathbb {R}^n$. Moreover, given a vector $v \in \mathbb {R}^n$, we will denote with $e^{v}$ the vector $(e^{v(i)})_{i=1}^n \in \mathbb {R}^n$. Similarly it will be useful to denote with $\log (v)$ the vector $(\log (v(i)))_{i=1}^n \in \mathbb {R}^n$.

## Kinetic Systems

In this section we recall the definition of kinetic systems and of some of their properties. This allows us to fix the notation that will be used later and to recall some properties that will be repeatedly used in the paper.

A kinetic system is a set of substances that interact via some chemical reactions, taking place at some given rates. In some cases, the topological properties of a kinetic system, determine the qualitative behaviour of the kinetic system, which turns out to be independent on the rates of the reactions. An example of a qualitative property, that in some cases depends only on the topological properties of the network is the property of detailed balance that holds for every linear chemical network that is a tree (see Feinberg (2019)). This is the reason why we start this section by introducing the concept of chemical network and later we will define kinetic systems as a chemical networks to which we associate a kinetics.

### Chemical Reaction Networks

In this section we give the definition of chemical networks and introduce some of their properties, for instance we provide the definition of conservative chemical networks and of bidirectional chemical networks. As we will see, a chemical network consists of a set of substances and a set of reactions and can be associated with a graph with vertices in $\mathbb {Z}^N$, where *N* is the number of substances in the network. The properties that we formulate for chemical reaction networks are independent on the reactions rates associated to the reactions in $\mathcal {R}$.

#### Definition 2.1

*(Chemical network)* Let $\Omega :=\{ 1, \dots , N \}$. Let $r \ge 1$ and $\mathcal {R}:= \{ R_1, \dots , R_r \}$ where $R_j \in \mathbb {Z}^N\setminus \{ 0\}$ for every $j \in \{ 1, \dots , r \}$. Then we say that $(\Omega , \mathcal {R} )$ is a chemical network.

The elements of $\Omega$ are the *substances* of the network, while $\mathcal {R}$ is the set of the *reactions* of the network. Notice that we assume that $R_j \in \mathbb {Z}^N, \ \forall R_j \in \mathcal {R}$. This means that the reactions that we consider take place between complexes that have a natural number of molecules of each substance. In this paper a complex is a group of molecules of some substances.

Let $R \in \mathcal {R}$ be a reaction. We define the following three sets

$$ I(R):=\{ i \in \Omega : R(i) <0 \}, \quad F(R):= \{ i \in \Omega : R(i) >0 \} \ \text { and } \ D(R):=I(R) \cup F(R). $$

Hence, the chemical reaction *R* transforms the chemicals in the set *I*(*R*) into the chemicals in the set *F*(*R*). The set *I*(*R*) is the set of the initial substances, while *F*(*R*) is the set of the final substances. We assume that $I(R) \cap F(R) = \emptyset$ for every $R \in \mathcal {R}$. Another assumption that we make is that every substance $i \in \Omega$ belongs to at least one reaction, i.e. for every $i \in \Omega$ there exists an $R \in \mathcal {R}$ such that $i \in D(R)$ and that $D(R)\ne \emptyset$ for every $R \in \mathcal {R}$ (otherwise we would just remove the substance *i* from the set of substances $\Omega$).

The set of the integer complexes that are accessible starting from the complex ${\textbf {0}}_N$ is the set $\mathcal {A}(0)$ defined as

2.1 $$\begin{aligned} \mathcal {A}(0)= \{ v \in \mathbb {Z}^N : v= \sum _{k=1}^r \lambda _k R_k \text { for some } \{ \lambda _k \}_{k=1}^r \subset \mathbb {Z} \}. \end{aligned}$$

Similarly, we can define also the set of the states accessible from a state $v_0 \in \mathbb {Z}^N$, this is

$$ \mathcal {A} (v_0):= v_0 + \mathcal {A}(0). $$

A chemical network $(\Omega , \mathcal {R} )$ can be viewed as a graph $\mathcal {G} = (E, V)$ with a countable number of vertices in $\mathbb {Z}^N$. Indeed, we can define the graph $\mathcal {G} =(V, E)$ where $V:=\mathcal {A}(0)$ and

$$ E:= \{ (v_1, v_2) \in V \times V : v_1 -v_2 \in \mathcal {R} \}. $$

We stress that this graph is directed.

#### Definition 2.2

*(Bidirectional chemical network)* A chemical network is *bidirectional* if for every $R \in \mathcal {R}$ we have that $- R \in \mathcal {R}$.

Notice that a bidirectional network induce an undirected graph (*V*, *E*) .

Given a network $(\Omega , \mathcal {R})$ we consider the set of non-reverse reactions $\mathcal {R}_s \subset \mathcal {R}$, obtained identifying each reaction *R* with the reversed reaction $- R$. More precisely, the set $\mathcal {R}_s \subset \mathcal {R}$ is defined as

2.2 $$\begin{aligned} \mathcal {R}_s := \{ R \in \mathcal {R} : - R \notin \mathcal {R} \} \cup \{ R \in \mathcal {R} \setminus \{ R \in \mathcal {R} : - R \notin \mathcal {R} \} : \min {I(R) } < \min {F(R)} \}. \end{aligned}$$

Hence we have if $R \in \mathcal {R}$ is non-reversible, i.e. $-R \notin \mathcal {R}$, then $R \in \mathcal {R}_s$. Instead if $R, - R \in \mathcal {R}$ only one of the two reactions belong to $\mathcal {R}_s$. As a consequence for every $R_{1}, R_{2 } \in \mathcal {R}_s$ we have $R_{1} \ne - R_{2}$. Notice that if the network $(\Omega , \mathcal {R})$ is bidirectional, then $|\mathcal {R}|$ is even. We associate to the set of the reactions $\mathcal {R}$ the matrix ${\textbf {R}} \in \mathbb {Z}^{ N \times |\mathcal {R}_s|}$ defined as

2.3 $$\begin{aligned} {\textbf {R}}_{j k } = R_k(j) \ \forall j \in \{1, \dots , N \}, \ \forall k \in \{ 1, \dots , |\mathcal {R}_s|\} \ \text { where } \ R_k \in \mathcal {R}_s. \end{aligned}$$

We refer to the matrix ${\textbf {R}}$ as the *matrix of the reactions* associated with the network $(\Omega , \mathcal {R})$.

We can now provide the notion of *cycles* of a chemical network $(\Omega , \mathcal {R})$.

#### Definition 2.3

*(Cycles of a chemical network)* Let $(\Omega , \mathcal {R})$ be a chemical network. Assume that ${\textbf {R}} \in \mathbb {Z}^{ N \times |\mathcal {R}_s|}$ is the matrix of the reactions. The space of the cycles of the chemical network $(\Omega , \mathcal {R})$ is defined as

$$ \mathcal {C} := \ker ({\textbf {R}}). $$

In particular, we say that a chemical network has no cycles if $\ker ({\textbf {R}}) = \{ {\textbf {0}}_{|\mathcal {R}_s| } \}$. It is possible to interpret the definition of cycles in terms of the cycles of the graph $\mathcal {G} = (V,E)$ as follows. The fact that $c \in \ker ({\textbf {R}})$ implies that there exists a sequence of reactions $\{ R_{i}\} _{\{i: c(i) \ne 0\}} \subset \mathcal {R}$ that, when applied to the composition ${\textbf {0}}_N$, produces the composition ${\textbf {0}}_N$, indeed by definition we have that

$$ \sum _{i=1}^{|\mathcal {R}|/2}c(i) R_{i}={\textbf {0}}_N. $$

Hence this means that there exists a cycle that contains the composition ${\textbf {0}}_N$ in the graph $\mathcal {G}$ defined above. The values $\{ |c(i)|\}_{i=1}^{|\mathcal {R}_s|}$ are the numbers of times that the reaction ${\textbf {R}} e_i=R_i \in \mathcal {R}$ appears in the cycle. In the following it is convenient to use the notation $R\in \mathcal {C}$ to indicate that there exists a $i\in \{1, \dots , |\mathcal {R}|/2 \}$ such that $R={\textbf {R}} e_i$ and such that there exists a $c \in \mathcal {C}$ with $c(i)\ne 0$. In order to clarify the notation introduced here we conclude this subsection with an example of chemical network that has cycles.

#### Example 2.4

Consider the chemical network $(\Omega , \mathcal {R})$ associated with the chemical reactions

$$ (1) \ \rightarrow \ (2) , \quad (2) +(2) \rightarrow \ (1)+(1) . $$

Notice that in this example we have one-directional reactions. The matrix of the reactions is

$$ {\textbf {R}}:= \left( \begin{array}{ccc} & -1 & 2 \\ & 1 & -2 \\ \end{array} \right) . $$

The space of the cycles is $\mathcal {C}:= \operatorname {span} \{ (2,1)\}$. If we apply twice the reaction $$R_1=\left( \begin{array}{c} -1 \\ 1 \\ \end{array} \right) $$ and once the reaction $$R_2= \left( \begin{array}{c} 2 \\ -2 \\ \end{array} \right) $$ to the composition (0, 0) we obtain again the composition (0, 0), indeed

$$ (0,0) \ \underset{R_1}{\rightarrow }\ (-1, 1) \ \underset{R_1}{\rightarrow }\ (-2, 2) \ \underset{R_2}{\rightarrow }\ (0,0) . $$

#### Conservative Networks

In this section we introduce the definition of conservative network. To this end we start by introducing the definition of stoichiometric subspace.

##### Definition 2.5

*(Stoichiometric subspace)* The stoichiometric subspace $\mathcal {S}$ of a reaction network $(\Omega , \mathcal {R})$ is

2.4 $$\begin{aligned} \mathcal {S} := \operatorname {span}\{ R: R \in \mathcal {R} \}. \end{aligned}$$

We recall that the chemical reactions $$R_i $$ are vectors in $$\mathbb {Z}^N$$. Since we are taking the span over the real numbers we have that $$\mathcal {S} \subset \mathbb {R}^N$$.

##### Definition 2.6

*(Stoichiometric compatibility classes)* Let $(\Omega , \mathcal {R})$ be a chemical network. Let $\mathcal {S}$ be the corresponding stoichiometric subspace. Two vectors $n, n' \in \mathbb {R}_+^N$ are stoichimetrically compatible if $n- n' \in \mathcal {S}$.

The stoichiometric compatibility is an equivalence relation. Given a vector $$v\in \mathbb {R}_+^N$$, the equivalence class $$[v]_\mathcal {S}$$ generated by *v* is defined as $$[v]_\mathcal {S}:=\{ x \in \mathbb {R}_+^N: x-v \in \mathcal {S} \} $$. Consider an initial vector $$n_0 \in \mathbb {R}_+^N$$ of concentration of substances in a chemical network $$(\Omega , \mathcal {R})$$. Let *n*(*t*) be the evolution of the concentration $$n_0 $$ due to the chemical reactions taking place in the network, then it holds that $$n(t)- n_0 \in \mathcal {S} $$. Therefore, independently on the reaction rates, *n*(*t*) will be stoichimetrically compatible with $$n_0$$ for every positive time $$t>0$$, i.e. $$n(t) \in [n_0]_{\mathcal {S}} $$.

We now explain that we can associate a set of conservation laws to a chemical network.

##### Definition 2.7

*(Set of conservation laws)* The set $\mathcal {M}$ of conservation laws of a chemical network $(\Omega , \mathcal {R})$ is defined as

2.5 $$\begin{aligned} \mathcal {M} := \mathcal {S}^\perp . \end{aligned}$$

Notice that, by definition, given a conservation law $m \in \mathcal {M}$ we have that

$$ m^T R = {\textbf {0}},\quad \forall R \in \mathcal {R}. $$

As a consequence the vector of the concentrations of substances at positive times, *n*(*t*) obtained as the evolution of an initial vector of concentrations $n_0$ is such that

$$ m^T n_0 = m^T n(t), \quad {\forall t >0,\ \forall m \in \mathcal {M}. } $$

This explains why we refer to $\mathcal {M}$ as the set of the conservation laws. Notice that by definition $\mathcal {M} \subset \mathbb {R}^N$, however, for physically relevant chemical networks we expect conservation laws to be non-negative. This motivates the following definition of the set $\mathcal {M}_+$ of non-negative conservation laws

$$ \mathcal {M}_+:=\mathcal {M} \cap \mathbb {R}_*^N . $$

Now that we introduced the definition of conservation law we can write the definition of conservative network.

##### Definition 2.8

*(Conservative chemical network)* We say that the network $(\Omega , \mathcal {R} )$ is conservative if

2.6 $$\begin{aligned} \mathcal {M}_+ \cap \mathbb {R}_+^N \ne \emptyset . \end{aligned}$$

In particular, a chemical network is conservative if and only if every substance $i \in \Omega$ appears in a conservation law as explained in the following lemma.

##### Lemma 2.9

Let $(\Omega , \mathcal {R})$ be a chemical network. Then the following statements are equivalent.

1. The chemical network is conservative.
2. For every $j \in \Omega$ there exists a $m \in \mathcal {M}_+$ such that $m(j) >0$.

##### Proof

Assume that 2. holds. Then for every $j \in \Omega$ there exists a $m_j \in \mathcal {M}$ such that $m_j(j) >0$. Therefore $m = \sum _{j \in \Omega } m_j \in \mathcal {M}$ is such that $m \in \mathbb {R}_+^N$ and therefore the network is conservative. The vice versa follows immediately by the fact that, by definition, there exists a $m \in \mathcal {M}_+ \cap \mathbb {R}_+^N$. $\square$

##### Lemma 2.10

Assume that the chemical network $(\Omega , \mathcal {R})$ is conservative. Then the set of the extreme rays of the positive cone $\mathcal {M}_+$ are a basis of $\mathcal {M}$.

##### Proof

Since the system is conservative there exists a vector $\overline{m} \in \mathcal {M}_+ \cap \mathbb {R}_+^N$. Let *V* be the vector subspace of $\mathcal {M}$ generated by $\overline{m}$. We define the following affine subspace $\mathcal {L}$ of $\mathcal {M}$ as $\mathcal {L}:= \overline{m} + V^\perp$. Let us define the set $\mathcal {D}:= \mathcal {L} \cap \mathcal {M}_+$. The set $\mathcal {D}$ is convex and compact by definition. Therefore Krein–Milman theorem (see Rockafellar (1970)) guarantees that the set $\mathcal {D}$ is the convex hull of its extremal points and the set of the extremal points of $\mathcal {D}$ is non-empty. Notice that the extremal points of $\mathcal {D}$ define the set of the extreme generators of the cone $\mathcal {M}_+$. The extreme generators are linear independent vectors, hence the number of extremal points is finite and they generate $\mathcal {M}_+$. The desired conclusion follows. $\square$

##### Example 2.11

Consider the chemical network corresponding to the reactions

$$ (1)+(2) \rightarrow (3)+(3), \quad (3) \rightarrow (2). $$

Notice that in this example we have one-directional reactions. Then $\Omega :=\{1, 2, 3 \}$ and the set of the reactions is

$$ \mathcal {R} := \left\{ \left( \begin{array}{c} - 1 \\ - 1 \\ 2 \end{array} \right) , \left( \begin{array}{c} 0 \\ 1 \\ - 1 \end{array} \right) \right\} . $$

Then $\mathcal {M} = \operatorname {span}\{ (1,1,1)^T \}$. Therefore the chemical network $(\Omega , \mathcal {R})$ is conservative. Instead, consider the chemical network induced by

$$ (1)+(2) \rightarrow (3), \quad (3) \rightarrow (2). $$

It is easy to see that $\mathcal {M} = \operatorname {span}\{ (0,1,1) \}$. Therefore the chemical network is not conservative.

### Kinetic Systems: Chemical Networks Endowed with a Rate Function

In this section we state the definition of kinetic systems. A kinetic system is a chemical network to which we associate a kinetics. In particular we will always consider mass action kinetics, hence it is enough to associate to the set of reactions a set of reactions rates.

A *reaction rate function* $\mathcal {K}: \mathcal {R} \rightarrow \mathbb {R}_+$ is a function that associate to each reaction its rate, i.e. $\mathcal {K}: R \mapsto \mathcal {K}(R)=:K_R \in \mathbb {R}_+$. We are now ready to give the definition of kinetic system.

#### Definition 2.12

*(Kinetic system)* A kinetic system $(\Omega , \mathcal {R}, \mathcal {K})$ is a chemical network $(\Omega , \mathcal {R} )$ endowed with the reaction rate function $\mathcal {K}$. The kinetic system $(\Omega , \mathcal {R}, \mathcal {K} )$ is bidirectional if the chemical network $(\Omega , \mathcal {R} )$ is bidirectional and is conservative if the chemical network $(\Omega , \mathcal {R} )$ is conservative.

We can associate to a kinetic system $(\Omega , \mathcal {R}, \mathcal {K} )$ a system of ODEs describing the evolution in time of the concentrations of species in the network, $n:=(n_1, \dots , n_N)^T \in \mathbb {R}_*^N$, i.e.

2.7 $$\begin{aligned} \frac{d n (t) }{dt} = \sum _{ R \in \mathcal {R} } K_R R \prod _{i\in I(R)} {(n_i)}^{-R(i)} , \quad n(0)=n_0 \in \mathbb {R}_*^N. \end{aligned}$$

In order to explain the interpretation of the terms in equation (2.7) it is convenient to write the evolution in time of the concentration $n_j$ of one substance $j \in \Omega$

$$\begin{aligned} \frac{d n_j (t) }{dt} = \sum _{ R \in \mathcal {R} } K_R R(j) \prod _{i\in I(R)} {(n_i)}^{-R(i)} , \quad n(0)=n_0 \in \mathbb {R}_*^N. \end{aligned}$$

The evolution of $n_j$ is driven by a loss and a gain term. The gain term is due to all the reactions that produce *j*, i.e. by all the reactions such that $i \in F(R)$, hence $R(j)>0$. The gain term due to each reaction is given by mass-action law, hence is given by $K_R R(j) \prod _{i \in I(R) } (n_i)^{-R(i)}$. The total gain due to all the reactions in the network is

$$ \sum _{ R \in \mathcal {R} : R(j) >0 } K_R R(j) \prod _{i\in I(R)} {(n_i)}^{-R(i)}. $$

Similarly, the loss term in *j* is due to the reactions that are such that $j \in I(R)$, hence $R(j) <0$. The contribution of the reaction *R* to the loss term due to the reaction *R* is given by $K_R R(j) \prod _{i \in I(R) } (n_i)^{-R(i)}$. The total loss is given by

$$ - \sum _{ R \in \mathcal {R} : R(j) <0 } K_R R(j) \prod _{i\in I(R)} {(n_i)}^{-R(i)}. $$

The following lemma is useful to rewrite the system of ODEs (2.7) in terms of the fluxes $$J_R $$ when the kinetic system is bidirectional.

#### Lemma 2.13

Assume that $(\Omega , \mathcal {R}, \mathcal {K})$ is a bidirectional kinetic system. Then, for every $n \in \mathbb {R}_*^N$ we have that

$$ \sum _{ R \in \mathcal {R} } K_R R \prod _{i \in I(R)} {(n_i)}^{-R(i)} = \sum _{ R \in \mathcal {R}_s } R J_R(n), $$

where the fluxes $$J_R (n) $$ are defined as

2.8 $$\begin{aligned} J_R (n):= K_R \prod _{ j \in I(R) } {(n_j)}^{-R(j)} - K_{- R } \prod _{ j \in F(R) } {n_j}^{R(j)}. \end{aligned}$$

#### Proof

Since $(\Omega , \mathcal {R}, \mathcal {K})$ is bidirectional, then we have that

$$\begin{aligned} \sum _{ R \in \mathcal {R} } K_R R \prod _{i \in I(R)} {(n_i)}^{-R(i)}&= \sum _{ R \in \mathcal {R}_s } K_R R \prod _{i \in I(R)} {(n_i)}^{-R(i)} - \sum _{ R \in \mathcal {R}_s } K_{-R} R \prod _{i \in I(-R)} {n_i}^{R(i)} \\&= \sum _{ R \in \mathcal {R}_s } R \left( K_R \prod _{i \in I(R)} {(n_i)}^{-R(i)} - K_{-R} \prod _{i \in F(R)} {n_i}^{R(i)} \right) \\&= \sum _{ R \in \mathcal {R}_s } R J_R(n). \end{aligned}$$

$$\square $$

As a consequence, when the kinetic system $(\Omega , \mathcal {R}, \mathcal {K})$ is bidirectional the system of ODEs (2.7) can be written as

2.9 $$\begin{aligned} \frac{dn(t)}{dt} = \sum _{ R \in \mathcal {R}_s } R J_R(n), \quad n(0)= n_0 \in \mathbb {R}_*^N. \end{aligned}$$

## Detailed Balance Property of Kinetic Systems

In this section we study one of the most important properties of kinetic systems: the property of detailed balance. From the mathematical point of view, an important consequence of the detailed balance property of kinetic systems is the existence of a unique positive stable steady state describing the long-time behaviour of the kinetic system. In this Section we state results that are standard, but we collect them here in a way that is convenient for our purposes.

### Detailed Balance Property

In this section, we give the definition of detailed balance property for kinetic systems and we state three conditions that are equivalent to the detailed balance property. The detailed balance property for bidirectional kinetic system states that each reaction is balanced by its reverse reaction at the steady state.

#### Definition 3.1

*(Detailed balance property)* A bidirectional kinetic system $(\Omega , \mathcal {R}, \mathcal {K} )$ satisfies the detailed balance property if there exists a $\overline{N} \in \mathbb {R}_+^N$ of (2.7) such that

3.1 $$\begin{aligned} K_R \prod _{i\in I(R)} {(\overline{N}_i)}^{-R(i) } = K_{-R} \prod _{i \in F(R) } {\overline{N}_i}^{R(i) }, \ \forall R\in \mathcal {R}_s. \end{aligned}$$

Notice that by the definition of detailed balance we have that $\overline{N}$ is such that $J_R(\overline{N}) =0$ for every $R \in \mathcal {R}$. Therefore $\overline{N}$ is a steady state of (2.7).

We now state conditions that are equivalent to the detailed balance property and that are useful in order to check if a kinetic system satisfies the detailed balance property or not.

#### Lemma 3.2

Let $(\Omega , \mathcal {R}, \mathcal {K} )$ be a bidirectional kinetic system. The following statements are equivalent.

1. The system satisfies the detailed balance property.
2. Condition (3.1) holds for every positive steady state of the system of ODEs (2.7) corresponding to $(\Omega , \mathcal {R}, \mathcal {K})$.
3. Let ${\textbf {R}}$ be the matrix of the reactions. For every cycle $c \in \mathcal {C}$ it holds that
  3.2 $$\begin{aligned} \prod _{ j=1}^{r/2} \left( \frac{K_{R_j}}{K_{-R_j}} \right) ^{c(j)} = 1, \end{aligned}$$
  here $$r=|\mathcal {R}|$$.

#### Proof

For the proof of the equivalence between 1 and 2 we refer to the proof of Theorem 14.2.1 in Feinberg (2019). For the equivalence between property 3 and 1 we refer to Feinberg (1989). $\square$

The condition 3. in Lemma 3.2 is often referred in the literature as *circuit condition* or *Wegscheider criterion* (see Wegscheider (1902)).

### Energy Associated to Kinetic Systems with Detailed Balance

In this section we explain that when a kinetic system satisfies the detailed balance property, then it is possible to associate to each chemical in the system a vector of energies. As will be explained later this energy determines the values of the steady states of the system of ODEs (2.7).

We start by associating an energy to each reaction taking place in the chemical network. Consider a kinetic system $(\Omega , \mathcal {R})$ that is bidirectional. We define the *Gibbs free energy function* associated to the set of reactions $\mathcal {R}$ as the function $\mathcal {E}: \mathcal {R} \rightarrow \mathbb {R}$ that maps each reaction to an energy as follows

$$ \mathcal {E} (R): = \log \left( \frac{K_{-R}}{ K_{R}} \right) . $$

The energy $\mathcal {E}(R)$ associated with the reaction $R \in \mathcal {R}$ describe the change of energy that takes place during the reaction *R*.

We extend the definition of the energy function $\mathcal {E}$ to sequences of reactions. To this end we identify the vectors $x \in \mathbb {R}^{|\mathcal {R}|/2}$ with the sequence $\{ ( x(i), R_i )\}_{i=1}^{|\mathcal {R}|/2}$ where $R_i = {\textbf {R}} e_i$ and where the number *x*(*i*) is the number of times that the reaction $R_i$ takes place. We define the energy function $\overline{\mathcal {E}}$ associated with a sequence of reactions as the function $\overline{\mathcal {E} }: \mathbb {R}^{|\mathcal {R}|/2} \rightarrow \mathbb {R}$ defined by

$$ \overline{\mathcal {E} }(x) := \sum _{i=1}^{|\mathcal {R}|/2} x(i) \mathcal {E} (R_i). $$

Notice that $\overline{\mathcal {E} }(x)$ is the sum of the energies of the reactions induced by the sequence *x*. Hence from the physical point of view, it is the total change in the energy of the system induced by the sequence of reactions *x*.

#### Lemma 3.3

Assume that $(\Omega , \mathcal {R}, \mathcal {K} )$ is a kinetic system that satisfies the detailed balance condition. Let $c \in \mathcal {C}$, then $\overline{\mathcal {E}}(c) =0$.

#### Proof

The statement follows from Lemma 3.2 and the definition of the map $\mathcal {E}$. Indeed

$$ \overline{\mathcal {E}}(c)= \sum _{j =1 }^{|\mathcal {R}|/2} c(j) \mathcal {E}(R_j) =0, $$

where for every $j \in \{1, \dots , |\mathcal {R}|/2\}$ we have that $R_j = {\textbf {R}} e_j$ and where ${\textbf {R}}$ is the matrix of the reactions. $\square$

Finally we extend the definition of energy to the complexes that are formed in the network, i.e. to the set of the complexes $\mathcal {A}(0)$ reachable starting from the complex ${\textbf {0}}_N$ via the reactions in the chemical network. Assume that $\xi \in \mathcal {A} (0)$ where we recall that $\mathcal {A}(0)$ is defined by (2.1). Then we have that there exists a $x \in \mathbb {R}^{|\mathcal {R}|/2}$ such that $\xi = {\textbf {R}} x$. We define the energy of the complexes in $\mathcal {A}(0)$ as the map $\tilde{\mathcal {E}}: \mathcal {A}(0) \rightarrow \mathbb {R}$ defined by

$$ \tilde{\mathcal {E}} (\xi ) := \overline{ \mathcal {E}}(x). $$

Lemma 3.3 guarantees that $\tilde{ \mathcal {E}}$ is well defined for kinetic systems that satisfy the detailed balance property as we explain in the following Lemma.

#### Lemma 3.4

Assume that $(\Omega , \mathcal {K}, \mathcal {R})$ is a kinetic system that satisfies the detailed balance property. Then the map $\tilde{\mathcal {E}}$ is well defined.

#### Proof

Assume that $\xi = {\textbf {R}} x_1 = {\textbf {R}} x_2$. This implies that $x_1-x_2 \in \mathcal {C}$, hence $\mathcal {E}(x_1)- \mathcal {E}(x_2)=\mathcal {E}(x_1-x_2)=0$ and the fact that $\tilde{\mathcal {E}}$ is well defined follows. $\square$

The energy $\tilde{\mathcal {E}}$ associated with the composition $\xi$ is just the change in the energy that is necessary to reach the composition $\xi$ starting from the composition 0. It is natural to expect that this energy is given by the sum of the energies of each of the chemicals in the composition $\xi$. In the following lemma we prove that this is the case. More precisely, we prove that if a kinetic system satisfies the detailed balance property, then the energy $\tilde{\mathcal {E}}$ associated with a complex is additive, more precisely, it can be written as the weighted sum of the energies of the different substances that appear in the complex, see (3.5).

#### Lemma 3.5

(Additive energy) Assume that the kinetic system $(\Omega , \mathcal {R}, \mathcal {K} )$ satisfies the detailed balance property. Let $w \in \mathbb {R}^{|\mathcal {R}|/2}$ be defined as

3.3 $$\begin{aligned} w(i)=\mathcal {E}(R_i), \ \forall \ i=1, \dots , |\mathcal {R}|/2 . \end{aligned}$$

Then there exists at least one solution $E \in \mathbb {R}^N$ to the equation

3.4 $$\begin{aligned} w = {\textbf {R}}^T E. \end{aligned}$$

Moreover, for every $\xi \in \mathcal {A}(0)$ it holds that

3.5 $$\begin{aligned} \tilde{\mathcal {E} } (\xi ) = \sum _{j=1}^L \xi (j) E (j) \end{aligned}$$

for every solution *E* to (3.4) and where we recall that $L= |\Omega |$.

#### Proof

The detailed balance property guarantees that there exists a $\overline{N} \in \mathbb {R}_+^N$ such that

$$ \ln (K_R ) - \sum _{i \in I(R) } R(i) \ln ( \overline{N}_i ) = \ln (K_{-R} ) + \sum _{i \in F(R) } R(i) \ln ( \overline{N}_i)\quad \forall R \in \mathcal {R} . $$

This implies that

$$ w = {\textbf {R}}^T \ln ( \overline{N}). $$

Hence $E=- \ln \overline{N}$ is a solution to (3.4).

Let $\xi \in \mathcal {A}(0)$. Hence $\xi = {\textbf {R}} x$ for some $x \in \mathbb {Z}^{|\mathcal {R}|/2}$. By the definition of $\overline{\mathcal {E} }$ we have that

$$ \tilde{\mathcal {E}} (\xi ) =\overline{\mathcal {E}}(x) = \sum _{i=1}^{|\mathcal {R}|/2} x(i) \mathcal {E}(R_i) = w^T x. $$

Now notice that if the vector $E\in \mathbb {R}^{N}$ satisfies (3.4), then

$$ \tilde{\mathcal {E} }(\xi )= w^T x = E^T {\textbf {R}} x =E^T \xi , \quad \forall x \in \mathbb {R}^{|\mathcal {R}|/2}. $$

$$\square $$

Notice that the solution to (3.4) is unique only if and only if $$\mathcal {M} =\{ 0\}$$, otherwise we have that if $$E_1 $$ is a solution to (3.4), then also the vector $$E_2 \in \mathbb {R}^L $$ defined as

$$ E_2(i) = E_1(i) + \sum _{j=1}^L \mu _j m_j(i), \quad \forall i \in \Omega , $$

where $\{ m_j\}_{j=1}^L$ with $m_j \in \mathbb {R}^N$ is a basis of $\mathcal {M}$ and where $\{ \mu _j \}_{j=1}^L$ are the chemical potentials, is also a solution to equation (3.4). In the following we will refer to each of the solutions of equation (3.4) as the *energies induced by the kinetic system* $(\Omega , \mathcal {R}, \mathcal {K} )$.

In the chemical literature there is usually a well defined vector of energies $$E \in \mathbb {R}^N $$. On the other hand, the equilibrium solution $$N=(N_k) $$ of a chemical system for which the detailed balance property holds is of the form $$N=(N_k)_k=e^{- E_k \pm \sum _{j=1}^L \mu _j m_j(k) } $$ where $$m_j $$ for $$j =1, \dots L $$ are the conserved quantities and $$\mu _j$$ are the so-called chemical potentials. It turns out that the vector of energies *E* can be defined up to the addition of the quantity $$\sum _{j=1}^L \mu _j m_j(k)$$. Due to this we will talk in the following of a whole class of vectors of energies, which can be defined up to the addition of the quantity $$\sum _{j=1}^L \mu _j m_j(k)$$. In the cases in which we need to use different vectors of Gibbs free energies *E* and $$\tilde{E}$$ for a given chemical network, we will need to take into account explicitly the role of the chemical potentials.

### Stability of the Steady States for Kinetic Systems with the Detailed Balance Property

An important consequence of the detailed balance property is the existence of a natural free energy function that guarantees the existence of a globally stable positive steady state. In this section we firstly recall that, when the detailed balance property holds, the steady states of (2.7) can be written as a function of the energies *E* introduced in Subsection 3.2. We then recall that a unique steady state exists in each stoichiometric compatibility class. Finally we recall the proof the stability of this steady state.

#### Lemma 3.6

Assume that the kinetic system $(\Omega , \mathcal {R}, \mathcal {K})$ satisfies the detailed balance property. Then $N^* =(N^*_i)_{i=1}^N$ is a steady state of the system of ODEs (2.7) if and only if

3.6 $$\begin{aligned} N^*_i= e^{- E(i)}, \quad i \in \{ 1, \dots , N\} \end{aligned}$$

where $E \in \mathbb {R}^N$ is an energy induced by the kinetic system $(\Omega , \mathcal {R}, \mathcal {K})$.

#### Proof

Lemma 3.2 implies that the detailed balance property holds if and only if every steady state $N^*$ of (2.7) satisfies

$$ 0= J_R (N^*)= K_R \prod _{ j \in I(R) } {N^*_j}^{-R(j)} - K_{- R } \prod _{ j \in F(R) } {N^*_j}^{R(j)}, \quad \forall R \in \mathcal {R}_s. $$

This holds if and only if

$$ \frac{K_R}{K_{-R}}= \frac{ \prod _{j \in F(R) }{N^*_j}^{R(j)} }{\prod _{j \in I(R) }{(N^*_j)}^{-R(j)} } = \prod _{j \in D(R) }{N^*_j}^{R(j)}, \quad \forall R \in \mathcal {R} . $$

Therefore $N^*$ is a steady state of (2.7) if and only if it satisfies

$$ 0 = \sum _{j \in \Omega }{ R(j) \ln (N^*_j) } + \ln (K_{-R}) - \ln (K_R), \quad \forall R \in \mathcal {R}. $$

As a consequence $N^*$ is a steady state of (2.7) if and only if it satisfies ${\textbf {R}}^T \ln (N^*) + w =0$ where the vector $w \in \mathbb {R}^{|\mathcal {R}|/2}$ is defined as in (3.3). Therefore $N^*$ is a steady state if and only if

$$ \ln (N^*) =- E $$

where *E* is a solution to (3.4). $\square$

The following following lemma provides a useful way to compute the fluxes when the detailed balance property holds.

#### Lemma 3.7

Let $$(\Omega , \mathcal {K}, \mathcal {R})$$ be a kinetic system that satisfies the detailed balance condition. Then for every $$ R \in \mathcal {R}$$ we have that the fluxes $$J_R $$ defined as (2.8) satisfy

3.7 $$\begin{aligned} J_R(n) =K_R \prod _{i \in I(R)} (n_i)^{- R(i) } \left( 1- \prod _{i\in \Omega } n_i^{ R(i) } e^{ R(i) E(i) } \right) , \ \forall n \in \mathbb {R}_*^N \end{aligned}$$

where $E \in \mathbb {R}^N$ is an energy of $(\Omega , \mathcal {R}, \mathcal {K} )$, i.e. is any solution to (3.4).

#### Proof

Lemma 3.6 guarantees that the vector $\overline{N} =e^{-E}$ is a steady state of (2.7) if and only if *E* satisfies ${\textbf {R}}^T E= w$. Hence for every $R \in \mathcal {R}_s$ we have that

3.8 $$\begin{aligned} \frac{K_{-R} }{K_{R} } = e^{ R^T E } \end{aligned}$$

As a consequence substituting this in (2.8) we obtain that

$$\begin{aligned}&J_R(n) = K_R \left( \prod _{i \in I(R)} (n_i)^{- R(i) } - \prod _{i \in F(R)} n_i^{ R(i) } \prod _{i\in \Omega } e^{ R(i)E(i) } \right) \\&\quad = K_R \prod _{i \in I(R)} (n_i)^{- R(i) } \left( 1- \prod _{i\in \Omega } n_i^{ R(i) } e^{ R(i)E(i) } \right) . \end{aligned}$$

$$\square $$

#### Proposition 3.8

Assume that the kinetic system $(\Omega , \mathcal {R}, \mathcal {K} )$ satisfies the detailed balance property. Assume that the system of ODEs (2.7) admits only positive steady states. Then the system of ODEs (2.7) has a unique positive steady state *N* in each stoichiometric compatibility class and the steady state is globally stable.

The proof of Proposition 3.8 can be found in Feinberg (2019). We review the main ideas behind that proof. Assume that the kinetic system $(\Omega , \mathcal {R}, \mathcal {K} )$ satisfies the detailed balance property. Then the function $F: \mathbb {R}_*^N \rightarrow \mathbb {R}$ defined as

3.9 $$\begin{aligned} F(n):= \sum _{j \in \Omega } n_j \left( \log \left( n_j e^{E(j) }\right) - 1 \right) \end{aligned}$$

where *E* is an energy of $(\Omega , \mathcal {R}, \mathcal {K} )$, is the total *free energy* associated with the kinetic system $(\Omega , \mathcal {R}, \mathcal {K})$. The *dissipation* $\mathcal {D}$ of free energy is defined as

3.10 $$\begin{aligned} \mathcal {D}(n):= - \partial _t F (n). \end{aligned}$$

It is easy to check, using also Lemma 3.7, that if a kinetic system satisfies the detailed balance property then

$$\begin{aligned} \mathcal {D}(n)&=- \sum _{j \in \Omega } \partial _t n_j \log ( n_j e^{ E(j) })= {- \sum _{j \in \Omega } \sum _{R \in \mathcal {R}_s } R(j) J_R(n) \log ( n_j e^{ E(j) }) }\\&{= - \sum _{R \in \mathcal {R}_s } J_R(n) \log \left( \prod _{j \in \Omega } n_j^{R(j)} e^{R(j) E(j) }\right) }\\&= { \sum _{R \in \mathcal {R}_s } K_R \prod _{i \in I(R)} (n_i)^{- R(i) } \left( \prod _{j\in \Omega } n_j^{ R(j) } e^{ R(j)E(j) } -1 \right) \log \left( \prod _{j \in \Omega } n_j^{R(j)} e^{R(j) E(j) }\right) .} \end{aligned}$$

Notice that $\mathcal {D}(n) \ge 0$ for every $n \in \mathbb {R}_*^N$ because the function $(x-1) \log (x) \ge 0$ for every $x \ge 0$. Hence kinetic systems that satisfy the detailed balance property have a non-increasing free energy. Moreover, we have that $\mathcal {D}(n)=0$ if and only if $n = e^{- E}$. As a consequence of Lemma 3.6, we have that the steady states are the only minimizers of the free energy. Since for each stoichiometric compatibility class it is possible to prove that there exists a unique steady state (see (Feinberg 2019, Proposition 13.A.1, Corollary 13.A.3)), the statement of Proposition 3.8 follows by noting that *F* is a radially unbounded Lyapunov functional.

Notice that the statement of Proposition 3.8 can be reformulated as follows. If the kinetic system $$(\Omega , \mathcal {R}, \mathcal {K} ) $$ satisfies the detailed balance property, then we have that for every initial concentration $$n_0 $$ there exists a unique positive steady state $$\overline{N}$$ such that $$m^T \overline{N} = m^T n_0 $$ for every $$m \in \mathcal {M} $$ and such that the solution to the system of ODEs (2.7) is such that $$\lim _{t \rightarrow \infty } n(t)=\overline{N}$$.

We conclude this section by introducing the definition of closed kinetic system. A closed kinetic system is a kinetic systems that does not exchange substances with the environment, hence satisfies the detailed balance condition and is conservative and does not contain sources and sinks. In this paper we say that a reaction is a *source* if $I(R)=\emptyset$ and is a *sink* if $F(R)=\emptyset$.

#### Definition 3.9

A kinetic system $(\Omega , \mathcal {R}, \mathcal {K} )$ is closed if it satisfies the detailed balance property, it is conservative and

3.11 $$\begin{aligned} I(R) \ne \emptyset \ \text { and } \ F(R) \ne \emptyset \quad \forall R \in \mathcal {R}. \end{aligned}$$

## Reduction of a Kinetic System

In this section we deal with kinetic systems that exchange substances with the environment. In particular, the assumption that we make in this section is that the time scale at which the exchange of chemicals between the kinetic systems and the environment takes place is much shorter than the time scale at which the reactions in the kinetic system take place. Hence we take the limit as $\alpha \rightarrow \infty$ in (8.1). This justifies the assumption that certain substances in the kinetic system have constant concentration values.

### Reduction of a Chemical Network

In this section we explain how we define the reduction of a chemical network. Before doing that assume that $v \in \mathbb {Z}^N$ is a vector and that $A \subset \Omega$, then we define the projection $\pi _A v \in \mathbb {R}^{|A|}$ as

$$ \pi _A v :=\left( v(i)\right) _{i \in A}=(v( \min (A)), \dots , v(\max A)). $$

#### Definition 4.1

Let $(\Omega , \mathcal {R})$ be a chemical network and let $U \subset \Omega$ be such that $U\ne \emptyset$ and let $V:= \Omega \setminus U$. Let $v = |V | = |\Omega \setminus U |$. Without loss of generality we assume that $U=\{ d+1, \dots ,N \}$ and $V = \{1, \dots , d \}$. The set of the *U-reduced reactions* ${ \mathcal {R}}_{V}$ is defined as as

$$ {\mathcal {R} }_{V} := \{ R \in \mathbb {Z}^{ |V|} \setminus \{ {\textbf {0}}_d\} : R= \pi _V \overline{ R}, \ \overline{ R } \in \mathcal {R} \}. $$

Then the chemical network $(V, {\mathcal {R}}_{V })$ is the *U-reduced chemical network* associated with the chemical network $(\Omega , \mathcal {R})$.

We denote with ${\textbf {R}}_{V}$ the matrix of the reactions associated with the set of reactions $\mathcal {R}_{V}$. Notice that the set of reactions $\mathcal {R}_{V}$ can contain reactions *R* such that $I(R)=\emptyset$ or such that $F(R)=\emptyset$.

#### Remark 4.2

If $(\Omega , \mathcal {R})$ is bidirectional, then also $(V, {\mathcal {R}}_{V} )$ is bidirectional.

We stress that the number of the reactions in $\mathcal {R}$ can be strictly larger than the number of the reduced reactions $\mathcal {R}_V$. This can take place due two different reasons. Indeed, two reactions $R_1, R_2 \in \mathcal {R}$ with $R_1 \ne R_2$ and $R_1 \ne - R_2$ can be projected to the same reaction, i.e. it can happen that $\pi _V R_1 = \pi _V R_2$. Another possibility, is that a reaction $R \in \mathcal {R}$ is projected to zero as $\pi _V R =0.$ We illustrate these two possibilities in the following example.

#### Example 4.3

Consider the following set of reactions

$$ (1) + (5) \leftrightarrows (2), \quad (1) + (6) \leftrightarrows (2), \quad (2) \leftrightarrows (3),\quad (1) \leftrightarrows (4), \quad (4)+ (5) \leftrightarrows (3), \quad (4) + (6) \leftrightarrows (3). $$

In this case $\Omega =\{1,2,3,4,5,6 \}$ and the matrix of the reactions is given by

$$ {\textbf {R}}= \left( \begin{array}{ccccccc} & -1 & -1 & 0 & -1 & 0 & 0 \\ & 1 & 1 & -1 & 0 & 0 & 0 \\ & 0 & 0 & 1 & 0 & -1 & -1 \\ & 0 & 0 & 0 & 1 & 1 & 1 \\ & -1 & 0 & 0 & 0 & 0 & 1 \\ & 0 & -1 & 0& 0 & 1 & 0 \end{array} \right) . $$

Assume that $U:=\{ 5,6 \}$. The reduced chemical network is $(V, \mathcal {R}_V )$ where $V:=\{ 1,2,3,4 \}$ and the matrix associated to this set of reactions as in (2.3) is given by

$$ {\textbf {R}}_V:=\left( \begin{array}{ccccc} & -1 & 0 & -1 & 0 \\ & 1 & -1 & 0 & 0 \\ & 0 & 1 & 0 & -1 \\ & 0 & 0 & 1 & 1 \end{array} \right) . $$

Notice that $\pi _V R_1 =\pi _V {\textbf {R}} e_1 = \pi _V R_2 =\pi _V {\textbf {R}} e_2$ as well as $\pi _V R_5 =\pi _V {\textbf {R}} e_5 = \pi _V R_6 =\pi _V {\textbf {R}} e_6$. Consider instead $\overline{U}=\{ 1, 4\}$, hence the matrix of the reduced reactions is

$$ {\textbf {R}}_{\overline{V}}= \left( \begin{array}{cccccc} & 1 & 1 & -1 & 0 & 0 \\ & 0 & 0 & 1 & -1 & -1 \\ & -1 & 0 & 0 & 0& 1 \\ & 0 & -1 & 0& 1 & 0 \end{array} \right) $$

Notice that $$ \pi _{\overline{V}} R_4 = \pi _{\overline{V} } {\textbf {R}} e_4 =0$$.

This example motivates the following definitions.

#### Definition 4.4

(1-1-reduced reaction and reaction with zero-*V*-reduction) Let $(\Omega , \mathcal {R})$ be a chemical network and assume that *U* and *V* are as in Definition 4.1. Let us consider the *U*-reduced chemical network $(V, \mathcal {R}_V )$ induced by the chemical network $(\Omega , \mathcal {R})$. We say that a reaction $R \in \mathcal {R}_V$ is a one-to-one reduced reaction if

$$ |\pi _V^{-1} (R) |=1 . $$

We say that a reaction $R \in \mathcal {R}$ has zero-*V*-reduction if $\pi _V R =0$.

Notice that $| \mathcal {R}_V |= | \mathcal {R} |$ if and only if every $R \in \mathcal {R}$ is not a reaction that has a zero-*V*-reduction and every reaction in $\mathcal {R}_V$ is a 1-1 reaction. Otherwise we have $|\mathcal {R}_V | < |\mathcal {R}|$.

Finally notice that the reduction described above decomposes the matrix of the reactions ${\textbf {R}}$ associated with $\mathcal {R}$ in two matrices $\pi _U {\textbf {R}}\in \mathbb {R}^{(N-d) \times s}$ and $\pi _V {\textbf {R}} \in \mathbb {R}^{d \times s }$ such that

4.1 $$\begin{aligned} {\textbf {R}}= \left( \begin{array}{c} \pi _V {\textbf {R}} \\ \pi _U {\textbf {R}}\\ \end{array} \right) . \end{aligned}$$

Here the matrix $\pi _V {\textbf {R}}$ is the matrix wich has, as columns, the projections of the columns of ${\textbf {R}}$, i.e. the columns of $\pi _V {\textbf {R}}$ are of the form $\pi _V ({\textbf {R}} e_i)$. We stress here that the matrix $\pi _V {\textbf {R}}$ is, in general, different from the matrix of the reduced reactions ${\textbf {R}}_V$. If every reaction in the reduced network is one-to-one and every reaction in the non reduced network does not have zero-*V*-reduction, then we have that the matrix $\pi _V {\textbf {R}} = {\textbf {R}}_V$.

### Reduction of the Cycles

In the following sections we will provide precise topological conditions on the chemical networks $(\Omega , \mathcal {R})$ and on the reduced chemical network $(V, \mathcal {R}_{V} )$ that guarantee that if the kinetic system $(\Omega , \mathcal {R}, \mathcal {K} )$ satisfies the detailed balance property, then also the reduced kinetic system satisfies the detailed balance property. More precisely, these are properties of the cycles $\mathcal {C}$ of the chemical system $(\Omega , \mathcal {R})$ and on the the cycles $\mathcal {C}_{V}$ of the reduced chemical network. This is the reason why in this section we study in detail the relation between $\mathcal {C}$ and $\mathcal {C}_V$.

**Fig. 1:**
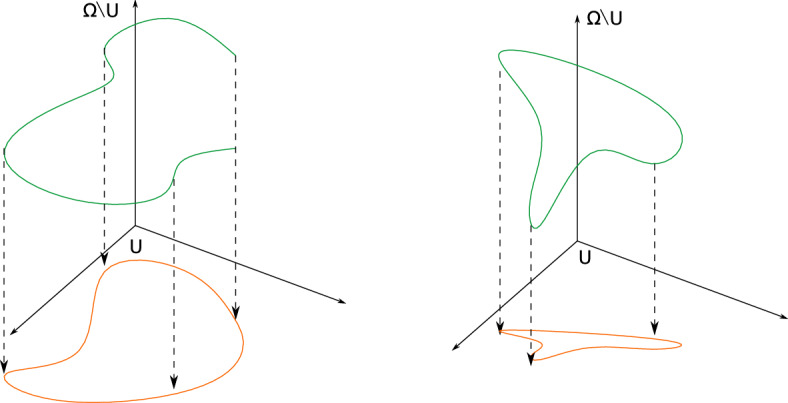
on the left we have a cycle in the reduced chemical network $(U, \mathcal {R}_V)$ that is not a cycle in the non-reduced chemical network $(\Omega , \mathcal {R})$. In the right we have a cycle in the reduced system that is also a cycle in the non reduced system

First of all we notice that if $|\mathcal {R}|= |\mathcal {R}_V |$ then we can compare directly the cycles and we have the following result. Notice that in this case we have that $\pi _V {\textbf {R}} ={\textbf {R}}_V$. The following lemma states that every cycle of the reduced chemical network is a cycle also in the non reduced chemical network. Moreover, in the following lemma we state conditions on the matrix ${\textbf {R}}$ that are necessary and sufficient to have that the cycles of the reduced system and the cycles of the non reduced system are the same.

#### Lemma 4.5

Let $(\Omega , \mathcal {R})$ be a chemical network with space of cycles $\mathcal {C}$. Let *U* and *V* be as in Definition 4.1 and let $(V, {\mathcal {R}}_{V})$ be the *U*-reduced network. Let $\mathcal {C}$ be the space of the cycles of the network $(\Omega , \mathcal {R})$ and let $\mathcal {C}_{V}$ be the space of cycles of the *U*-reduced network $(V, {\mathcal {R}}_{V})$. Assume that $|\mathcal {R}_V | = |\mathcal {R}|$. Then

4.2 $$\begin{aligned} \mathcal {C} \subset {\mathcal {C}}_{V}. \end{aligned}$$

Moreover, we have that $\mathcal {C} = {\mathcal {C} }_{V }$ if and only if $\ker ({\textbf {R}}_V)\subset \ker (\pi _U {\textbf {R}} )$, where $\pi _U {\textbf {R}} \in \mathbb {R}^{(L-d) \times |\mathcal {R}|/2 }$ is given by (4.1).

#### Proof

First of all we prove that $\mathcal {C} \subset {\mathcal {C}}_{V }$. Let $c \in \mathcal {C}$. By definition we have that $\sum _{i=1}^{s} c(i) R_i =0$ where $\mathcal {R}_i ={\textbf {R}} e_i$ and where $s =|\mathcal {R}_s|$ where we recall that the set $\mathcal {R}_s$ is defined as (2.2). By the definition of $\pi _V$ this implies that $\sum _{i=1}^{s} c(i) \pi _V R_i= 0$. Hence (4.2) follows.

Assume now that ${\mathcal {C} }_{V }\subset \mathcal {C}$. Hence, by the definition of $\pi _U {\textbf {R}}$, it follows that $\pi _U {\textbf {R}} c =0$ for every $c \in \mathcal {C}_{V } = \ker (\pi _V {\textbf {R}} )= \ker ( {\textbf {R}}_V )$. Hence $\ker ({\textbf {R}}_V )\subset \ker (\pi _U {\textbf {R}} )$. Assume instead that $\mathcal {C}_{V } = \ker ( {\textbf {R}}_V )\subset \ker (\pi _U {\textbf {R}} )$. Hence for every $c \in \mathcal {C}_{V }$ we have that $\pi _U {\textbf {R}} c =0$. As a consequence we have that $c \in \mathcal {C}$. $\square$

We have a similar statement when $|\mathcal {R} | > |\mathcal {R}_V|$. To formulate this statement it is convenient to introduce the definition of a suitable projection of cycles of the non reduced chemical network $(\Omega , \mathcal {R})$ to the reduced chemical network $(V, \mathcal {R}_V)$. To this end notice that if $c \in \mathcal {C}$, then it holds that

$$ \pi _V\left( \sum _{i=1}^s R_i c(i)\right) =\sum _{i=1}^s (\pi _V R_i) c(i) =0, $$

where $R_i = {\textbf {R}} e_i$ and $s=|\mathcal {R}_s|$. Moreover,

$$ \sum _{i=1}^s (\pi _V R_i ) c(i) = \sum _{j=1 }^v \overline{R}_j \sum _{ \{ i: \overline{R}_j = \pi _V R_i \} } c(i) $$

where $\overline{R}_j={\textbf {R}}_V e_i$ and *v* is the number of reactions in $(\mathcal {R}_V)_s$ where the set $(\mathcal {R}_V)_s$ is defined as in (2.2) with respect to $\mathcal {R}_V$. This implies that the vector $p[c] \in \mathbb {R}^{v}$ defined as

4.3 $$\begin{aligned} p[c](j):= \sum _{ \{ i: \overline{R}_j = \pi _V R_i \} } c(i), \quad j \in \{ 1, \dots , v\} \end{aligned}$$

is such that ${\textbf {R}}_V p[c]=0$. Therefore $p[c] \in \mathcal {C}_V$ is the projection of the cycle $c \in \mathcal {C}$. To clarify the above definition we make the following example.

#### Example 4.6

Recall the chemical network $(\Omega , \mathcal {R})$ of Example 4.3. Notice that ${\textbf {R}} (1,1,2,-2,1,1) =0$. Consider the reduction with respect to $U:=\{5,6\}$. We now apply the projection map to the cycle $c=(1,1,2,-2,1,1)$ and obtain that $p[c]=(2,2,-2,2)$. Notice that this is a cycle of the reduced system. Indeed

$$ {\textbf {R}}_V p[(1,1,2,-2,1,1)]= \left( \begin{array}{ccccc} -1 & 0 & -1 & 0 \\ 1 & -1 & 0 & 0 \\ 0 & 1 & 0 & -1 \\ 0 & 0 & 1 & 1 \end{array} \right) \left( \begin{array}{c} 2\\ 2 \\ -2 \\ 2 \end{array} \right) =0. $$

We are now ready to state the following Lemma 4.7, which is the analogous of Lemma 4.5 when we have that $|\mathcal {R} |> |\mathcal {R}_V |$.

#### Lemma 4.7

Let $(\Omega , \mathcal {R})$ be a chemical network with space of cycles $\mathcal {C}$. Let *U* and *V* be as in Definition 4.1 and let $(V, {\mathcal {R}}_{V})$ be the corresponding *U*-reduced network. Let $\mathcal {C}_{V}$ be the space of cycles of $(V, {\mathcal {R}}_{V})$. Then $p (\mathcal {C}):=\{ p[c]:c \in \mathcal {C} \} = {\mathcal {C} }_{V }$ if $\ker ( \pi _V{\textbf {R}} )\subset \ker (\pi _U {\textbf {R}})$, where $\pi _V{\textbf {R}} \in \mathbb {R}^{d \times |\mathcal {R}|/2 }$ and $\pi _U {\textbf {R}} \in \mathbb {R}^{(N-d) \times |\mathcal {R}|/2 }$ are defined as in (4.1).

#### Proof

The fact that $p(\mathcal {C}) \subset \mathcal {C}_{V}$ follows by the definition of the map *p*. We consider $c \in \mathcal {C}_{V }$ and assume that $\ker ( \pi _V {\textbf {R}}) \subset \ker ( \pi _U {\textbf {R}})$. Let $\tilde{c} \in \mathbb {R}^{|\mathcal {R}|}$ be such that $\tilde{c}(i) = c(i)$ for every $i \in \{ 1, \dots , |\mathcal {R}_V|\}$ and otherwise set $\tilde{c} (i)=0$. Notice that, upon reordering the reactions, by construction we have that $\pi _V{\textbf {R}} \tilde{c} =0$. Hence ${\textbf {R}} \tilde{c} =0$ and $\tilde{c} \in \mathcal {C}$. Notice also that $c=p[\tilde{c}]$ hence the desired conclusion follows. $\square$

We conclude this section by analysing the relation between the mass conservation laws of the chemical network $(\Omega , \mathcal {R})$ and of the *U*-reduced chemical network $(V, \mathcal {R}_{V})$.

#### Lemma 4.8

Let $(\Omega , \mathcal {R})$ be a chemical network with space of conservation laws $\mathcal {M}$. Let *U* and *V* be as in Definition 4.1 and let $(V, {\mathcal {R}}_{V})$ be the corresponding *U*-reduced network. Let $\mathcal {M}_{V}$ be the space of conservation laws of the *U*-reduced network $(V, {\mathcal {R}}_{V})$. Then every $m \in \mathcal {M}_{V}$ is such that

4.4 $$\begin{aligned} \left( \begin{array}{c} m \\ {\textbf {0}}_{N-d} \end{array}\right) \in \mathcal {M}. \end{aligned}$$

### Reduction of a Kinetic System

In the previous section we explained how to reduce a chemical network $(\Omega , \mathcal {R})$ to the network $(V, \mathcal {R}_V)$ where *V* is as in Definition 4.1. We now explain how to construct the corresponding reduced kinetic system.

#### Definition 4.9

Let $(\Omega , \mathcal {R}, \mathcal {K} )$ be a kinetic system. Let *U*, *V* be as in Definition 4.1. Let $n_U=(n_i)_{i \in U}$ be given. Consider the *U*-reduced kinetic system $(V, \mathcal {R}_V)$. We associate to $(V, \mathcal {R}_V)$ the rate reaction function ${\mathcal {K} }[n_U]: {\mathcal {R}}_{V} \rightarrow \mathbb {R}$ where

4.5 $$\begin{aligned} {\mathcal {K} }[n_U](R) = K_R[n_U] = \sum _{ \{ \overline{R} = \pi _V^{-1} (R) \} } \hat{K}_{\overline{R}} \end{aligned}$$

where

4.6 $$\begin{aligned} \hat{K}_{\overline{R} } := K_{\overline{R}} \prod _{s \in U \cap I({\overline{R}}) } n_s^{-{\overline{R}}(s)}. \end{aligned}$$

The kinetic system $(V, \mathcal {R}_{V}, {\mathcal {K} }[n_U])$ is the reduced kinetic system associated with the kinetic system $(\Omega , \mathcal {R}, \mathcal {K})$ where the concentrations of the substances in *U* are constant and equal to $n_{ U }$.

#### Remark 4.10

Assume that $(V, \mathcal {R}_{V}, {\mathcal {K} }[n_U])$ is the reduced kinetic system associated with the kinetic system $(\Omega , \mathcal {R}, \mathcal {K})$. If $\pi _V R\in \mathcal {R}_V$ is a 1-1 reduction, then the identity (4.5) implies that

4.7 $$\begin{aligned} \frac{K_{- \pi _V R}[n_U]}{K_{\pi _V R}[n_U]} = \frac{K_{- R}}{K_R } \prod _{s \in U} n_s^{{R}(s)} . \end{aligned}$$

## The Detailed Balance Property of Reduced Kinetic Systems

The aim of this section is to study when a reduced kinetic system inherits the detailed balance property of the non reduced kinetic system $(\Omega , \mathcal {R}, \mathcal {K} )$. In Subsection 5.1 we prove that if the frozen concentrations that appear in the cycles of the non reduced kinetic system are chosen at equilibrium values, then the reduced kinetic system satisfies the detailed balance property. In Subsection 5.2 instead we prove that the reduction of a kinetic system that satisfies the detailed balance property "in general" does not satisfy the detailed balance property. We stress that the question of understanding under which condition a given chemical reaction network with detailed balance reduces to a chemical network that still satisfies the detailed balance property is also studied in Polettini and Esposito (2014).

### Reduced Kinetic System with Some Concentrations Fixed at Equilibrium Values

In this section we provide sufficient conditions on $$n_U $$ that guarantee that if the kinetic system $$(\Omega , \mathcal {R}, \mathcal {K} )$$ satisfies the detailed balance property, then the reduced kinetic system $$(V, \mathcal {R}_V, \mathcal {K} [n_U])$$ satisfies the detailed balance condition. The main idea is that if the vector of the frozen concentrations $$n_U $$ is chosen at equilibrium concentrations, i.e. it is given by the projection on *U* of a steady state *N* of the system of ODEs (2.7) induced by the kinetic system $$(\Omega , \mathcal {R}, \mathcal {K}) $$, then the reduced kinetic system $$(V, \mathcal {R}_V, \mathcal {K}[n_U] ) $$ satisfies the detailed balance property. However this statement can be made stronger, as it is sufficient to have that the concentration of the substances in *U* are selected at equilibrium values if these substances appear in the cycles of the reduced system.

More precisely, we find that if every reaction that belongs to a cycle of the reduced kinetic system contains exactly the same substances as the corresponding non reduced reaction, i.e. if the substances in *U* do not appear in the reactions whose reduction belong to a cycle, then the detailed balance property of the reduced kinetic system follows directly by the detailed balance of the non reduced kinetic system for every value of frozen concentrations $n_U$. Instead, if we assume that some of the substances in the set *U* appear in some of the reactions in $\mathcal {R}$ whose reduction belong to a cycle, then selecting the concentrations of these substances at equilibrium values guarantees that the reduced system satisfies the detailed balance property.

In order to state these two results precisely it is useful to introduce the definition of the set $\mathcal {D}(\mathcal {C}_V)$. This is the set of the substances in $\Omega$ belonging to a reaction $R \in \mathcal {R}$ whose projection belongs to a cycle of the reduced system. More precisely, given a kinetic system $(\Omega , \mathcal {R}, \mathcal {K})$ and its reduction $(V, \mathcal {R}_V, \mathcal {K}[n_U])$ with space of cycles $\mathcal {C}_V$ we define the following set

5.1 $$\begin{aligned} \mathcal {D}(\mathcal {C}_V ):=\{ i \in \Omega : \exists R \in \mathcal {R} \quad \pi _V R \in \mathcal {C}_V, \ R(i)\ne 0 \}. \end{aligned}$$

#### Proposition 5.1

Assume that the kinetic system $(\Omega , \mathcal {R}, \mathcal {K} )$ satisfies the detailed balance property. Let $U \subset \Omega$ and $V:= \Omega \setminus U$. Consider the reduced kinetic system $(V, \mathcal {R}_{V}, \mathcal {K} [n_U] )$ where $n_U:=\{ n_s \}_{ s \in U }$ with $n_s>0$ for every $s \in U$.

1. If $U \cap \mathcal {D}(\mathcal {C}_V)= \emptyset$, then the kinetic system $(V, \mathcal {R}_{V}, \mathcal {K} [n_U] )$ satisfies the detailed balance property.
2. If $$ U \cap \mathcal {D}(\mathcal {C}_V) \ne \emptyset $$ and
  5.2 $$\begin{aligned} n_s = e^{- E(s) }, \quad \forall s \in U \cap \mathcal {D}(\mathcal {C}_V) \end{aligned}$$
  for an energy $E \in \mathbb {R}^N$ of the kinetic system $(\Omega , \mathcal {R}, \mathcal {K} )$. Then the kinetic system $(V, \mathcal {R}_{V}, \mathcal {K} [n_U] )$ satisfies the detailed balance property.

#### Proof

We start by proving 1. The fact that $U \cap \mathcal {D}(\mathcal {C}_V) = \emptyset$ implies that for every $R \in \mathcal {R}$ such that $\pi _V R \in \mathcal {C}_V$ it holds that $\pi _U R =0$. This implies that every reaction in $\mathcal {C}_V$ is a 1-1 reaction. Moreover, the fact that for every $R \in \mathcal {R}$ such that $\pi _V R \in \mathcal {C}_V$ it holds that $\pi _U R =0$ implies that $\ker ( \pi _V {\textbf {R}} ) \subset \ker ( \pi _U {\textbf {R}} )$. Indeed, let $x \in \ker ( \pi _V {\textbf {R}})$. Then by the definition of the projection *p* we have that $p[x] \in \mathcal {C}_V$. Since the reactions in $\mathcal {C}_V$ are 1-1 we have that, upon reordering the reactions, $x =(p[x], {\textbf {0}}_{r-v} )$ where we are using the notation $r= |\mathcal {R}|$ and $v= |\mathcal {R}_V |$. Notice that since $\mathcal {C}_V \cap U = \emptyset$, then

$$ \pi _U {\textbf {R}} x = \sum _{i=1 }^{r/2} x(i) \pi _U {\textbf {R}} e_i = \sum _{i=1 }^{v/2} p[x](i) \pi _U R_i =0. $$

Hence Lemma 4.7 implies that $p(\mathcal {C})=\mathcal {C}_V$. To conclude the proof notice that the fact that $U \cap \mathcal {D}(\mathcal {C}_V) = \emptyset$ implies the rates of the reactions in the cycles are not modified. As a consequence for every $c \in p(\mathcal {C} )= \mathcal {C}_V$ it holds that

$$ \sum _{j=1}^{v/2 } c(j) \mathcal {E} \left( R_j \right) = \sum _{j=1}^{v/2} c(j) \mathcal {E} \left( \pi _V R_j \right) =0 $$

where $v=|\mathcal {R}_V|$ and where we are using the fact that since the reactions in $\mathcal {C}_V$ are 1-1 then $p[c](i)=c(i)$ for every $i \in \{1, \dots , v\}$.

We now prove 2. Consider $\overline{R} \in \mathcal {R}_V$ such that $\overline{R} \in \mathcal {C}_V$ and there exists a $R \in \pi _V^{-1} (\overline{R})$ with $D(R) \cap U \ne \emptyset$. Let us compute $\frac{\hat{K}_{-R}}{\hat{K}_{R}}$ where $\hat{K}$ is defined as in (4.6). Using the fact that $n_U$ satisfies (5.2) with respect to a solution *E* of (3.4) we deduce that

$$\begin{aligned} \frac{\hat{K}_{-R}}{\hat{K}_{R}} = \frac{ K_{-R} \prod _{i \in U \cap F(R) } n_i^{R(i) }}{ K_{R} \prod _{i \in U \cap I(R) } n_i^{-R(i) }} = \frac{ K_{-R}}{K_R}\prod _{i \in U } n_i^{R(i) } = \frac{ K_{-R}}{K_R} \exp \left( - \sum _{i \in U } E(i)R(i) \right) . \end{aligned}$$

Therefore we have that for $\overline{R} \in \mathcal {R}_V$ it holds that

$$\begin{aligned} \mathcal {E}( \overline{R} )&= \log \left( \frac{ \sum _{R \in \pi ^{-1}_V (\overline{R}) } \hat{K}_{-R}}{\sum _{R \in \pi ^{-1}_V (\overline{R}) } \hat{K}_{R}}\right) \\&= \log \left( \frac{ \sum _{R \in \pi ^{-1}_V (\overline{R}) } \hat{K}_{R} \exp \left( \mathcal {E} (R) - \sum _{i \in U } E(i)R(i) \right) }{\sum _{R \in \pi ^{-1}_V (\overline{R}) } \hat{K}_{R}}\right) \\&=\log \left( \frac{ \sum _{R \in \pi ^{-1}_V (\overline{R}) } \hat{K}_{R} \exp \left( \sum _{i \in \Omega } E(i) R(i) - \sum _{i \in U } E(i) R(i) \right) }{\sum _{R \in \pi ^{-1}_V (\overline{R}) } \hat{K}_{R}}\right) \\&= \sum _{i \in V } E(i) \overline{R}(i). \end{aligned}$$

In the third equality we used the detailed balance property of $(\Omega , \mathcal {R}, \mathcal {K})$ and in the last equality the fact that $R(i)=\overline{R}(i)$ for every $\overline{R} \in \pi _V^{-1} (R)$ and for every $i \in V$. Consider now a $c \in \mathcal {C}_V$, then since ${\textbf {R}}_V c =0$ we have that

$$ \sum _{j=1}^{v/2} c(j) \mathcal {E}( \overline{R}_j ) = c^T {\textbf {R}}_V^T \pi _V E =0. $$

As a consequence the kinetic system $(V, \mathcal {R}_{V}, \mathcal {K} [n_U] )$ satisfies the detailed balance condition. $\square$

Proposition 5.1 has a consequence for systems with fluxes in a unique substance $s \in \Omega$. Clearly the statement of Proposition 5.1 holds in the case in which $|U|=1$. However when $U \cap \mathcal {D}(\mathcal {C}_{\Omega \setminus \{ s\} } ) \ne \emptyset$ and $\mathcal {M} \ne \{ 0\}$ it is possible to find for every value of $n_s >0$ an energy *E* that is such that $n_s = e^{-E(s)}$. This is the content of the following corollary.

#### Corollary 5.2

Let $(\Omega , \mathcal {R}, \mathcal {K} )$ be a kinetic system that satisfies the detailed balance property. If $\mathcal {M} \ne \{ 0\}$, then there exists a $s \in \Omega$ such that the reduced system $(V, \mathcal {R}_{V },\mathcal {K}[n_s])$, with $V:= \Omega \setminus \{ s \}$, satisfies the detailed balance condition for any value of $n_s>0$.

#### Proof

Since we assume that $$\mathcal {M} \ne \{ 0\} $$, then there exists a $$s \in \Omega $$ such that $$m(s) \ne 0 $$ for some $$m \in \mathcal {M} $$. Therefore, to conclude the proof we need to prove that, for any value of $$n_s $$, there exists a vector $$E \in \mathbb {R}^{N}$$ such that $${\textbf {R}}^T E = w $$ and $$ E(s)= -\log (n_s) $$. Assume by contradiction that $$E(s) \ne -\log (n_s)$$ for every *E* that satisfy $${\textbf {R}}^T E = w $$. Since there exists a $$m \in \mathcal {M} $$ such that $$m(s)\ne 0 $$ we can define the vector $$v:= \frac{\log (n_s) + E(s)}{m(s)} m $$. Define the vector $$E^*:= E- v $$, then

$$ R^T E^* = R^T E - R^T v =w \text { and } E^*(s)= - \log (n_s). $$

This is a contradiction, therefore there exists a vector $E \in \mathbb {R}^{N}$ such that ${\textbf {R}}^T E = w$ and $E(s)= -\log (n_s)$. As a consequence the assumptions of Proposition 5.1 hold, hence the system with fluxes $(\Omega , \mathcal {R}, \mathcal {K}, n_s)$ satisfies the detailed balance property. $\square$

#### Remark 5.3

Notice that Proposition 5.1, combined with 3.8, has in important consequence. Indeed if we assume that the set of the constant concentrations $$n_U $$ satisfy (5.2), then the system of ODEs (2.7) corresponding to the reduced kinetic system $$(V, \mathcal {R}_V, \mathcal {K} [n_U]) $$ has a unique steady state for every stoichiometric compatibility class. This steady state is globally stable. Notice that the compatibility class here is with respect to the stoichiometry $$\mathcal {S}_V$$. Similarly, Corollary 5.2 implies that under the assumption of the corollary we have that the reduced kinetic system $$(V, \mathcal {R}_V, \mathcal {K} [n_s]) $$ has a unique steady state for every compatibility class. This steady state is globally stable.

### Stability of the Detailed Balance Property of the Reduced System

In this section we study under which topological condition the reduced kinetic system $(V, \mathcal {R}_V, \mathcal {K} [n_U])$ inherits the detailed balance property of the original kinetic system $(\Omega , \mathcal {R}, \mathcal {K})$ independently on the choice of the values of the frozen concentrations $n_U$.

#### Theorem 5.4

Assume that the kinetic system $(\Omega , \mathcal {R}, \mathcal {K} )$ satisfies the detailed balance property. Let $\mathcal {C}$ be the associated space of cycles. Let *U* and *V* be as in Definition 4.1 and let $(V, \mathcal {R}_{V}, {\mathcal {K} }[n_U])$ be the *U*-reduced kinetic system $(V, \mathcal {R}_{V}, {\mathcal {K} }[n_U])$. Let $\mathcal {C}_V$ be the space of the cycles associated with $(V, \mathcal {R}_{V}, {\mathcal {K} }[n_U])$. Assume that

1. each reaction $R \in \mathcal {C}_V$ is 1-1 (see Definition 4.4);
2. $\mathcal {C}$ does not contain reactions with zero-*V*-reductions (see Definition 4.4);
3. $p( \mathcal {C}) = \mathcal {C}_{V}$, where we recall that the projection *p* is defined as in (4.3).

Then $(V, \mathcal {R}_{V}, {\mathcal {K} } [n_U] )$ satisfies the detailed balance property for every $n_U = {( n_i )}_{ i \in U }$.

Notice that the three conditions in the above theorem imply that all the cycles of the reduced and of the non reduced chemical systems are as in the right part of Figure 1.

#### Proof

Since we are assuming that every $$\overline{R} \in \mathcal {C}_V $$ is 1-1 we have that for every $$\overline{R}_j \in \mathcal {C}_V $$ there exists a unique $$R_j $$ such that $$\pi _V R_j=\overline{R}_j$$. Moreover since equality (4.7) holds, we deduce that

$$\begin{aligned} &  \mathcal {E}( \pi _V R_j)= \log \left( \frac{ K_{- \pi _V R_j} [n_U] }{ K_{\pi _V R_j} [n_U]} \right) = \log \left( \frac{ K_{ - R_j} }{ K_{R_j}} \right) \\ &  \quad - \sum _{s \in U } R_j(s) \log ( n_s) = \mathcal {E}(R_j) - \sum _{s \in U } R_j(s) \log ( n_s). \end{aligned}$$

Therefore for every $c \in \mathcal {C}_V$ we have that

$$ \sum _{j =1}^{v/2} c(j) \mathcal {E}(\pi _V R_j)= \sum _{j =1}^{v/2} c(j) \mathcal {E}(R_j) - \sum _{j =1}^{v/2} c(j) \sum _{s \in U } R_j(s) \log ( n_s) $$

where $v:=|\mathcal {R}_V|$. We now use the fact that $p(\mathcal {C})=\mathcal {C}_V$ to deduce that there exists a $\overline{c} \in \mathcal {C}$ such that $p(\overline{c})=c$. Since $\mathcal {C}$ does not contain reactions with zero-*V*-reduction and $\mathcal {C}_V$ does not contain reactions that are not 1-1 reactions, we deduce that (upon reordering of the reactions) the cycle $\overline{c}$ has the following form $\overline{c}=({\textbf {0}}, c)$. Since $(\Omega , \mathcal {R}, \mathcal {K} )$ satisfies the detailed balance property it follows that

$$\begin{aligned} 0= \sum _{j =1}^{r/2} \overline{c} (j) \mathcal {E}(R_j) = \sum _{j =1}^{v /2} c(j) \mathcal {E}(R_j) \end{aligned}$$

where $r= |\mathcal {R}|$. Therefore we deduce that

$$ \sum _{j =1}^{r/2} c(j) \mathcal {E}(\pi _V R_j)= - \sum _{s \in U } \sum _{j =1}^{r/2} c(j) R_j(s) \log ( n_s) =0. $$

Where the last equality comes from the fact that $\overline{c} \in \mathcal {C}$, hence $\sum _{j =1}^{r/2} \overline{c}(j) R_j(s) =0$ for every $s \in U$. $\square$

#### Remark 5.5

Notice that the requirements 1., 2. and 3. in Theorem 5.4 are all topological requirements that depend only on the chemical network $(\Omega , \mathcal {R})$ and do not depend on the choice of the rate function. As a consequence, when the assumptions of Theorem 5.4 are satisfied, then the detailed balance property of the reduced kinetic system is stable under perturbations of the reaction rates of the non reduced kinetic system $(\Omega , \mathcal {R}, \mathcal {K} )$.

In Theorem 5.4 we have three assumptions. These three assumptions are all needed in order to have a reduced kinetic system that satisfies the detailed balance property in a stable manner. We start by analysing what happens when assumptions 1. and 2. of Theorem 5.4 holds, but $p (\mathcal {C}) \ne \mathcal {C}_V$.

Since in the following proposition we deal with a perturbation of the rates it is convenient to introduce the definition of norm of a rate function. Let $\mathcal {K}$ be a rate function, then

5.3 $$\begin{aligned} \Vert \mathcal {K} \Vert := \max _{R \in \mathcal {R}} |\mathcal {K} (R) | . \end{aligned}$$

#### Proposition 5.6

Assume that the kinetic system $(\Omega , \mathcal {R}, \mathcal {K} )$ satisfies the detailed balance property. Let $\mathcal {C}$ be the associated space of cycles. Let *U* and *V* be as in Definition 4.1 and let $(V, \mathcal {R}_{V}, {\mathcal {K} }[n_U])$ be the *U*-reduced kinetic system $(V, \mathcal {R}_{V}, {\mathcal {K} }[n_U])$. Assume that $p(\mathcal {C}) \ne \mathcal {C}_{V}$ and each reaction in $\mathcal {C}_V$ is 1-1 and $\mathcal {C}$ does not contain reactions with zero-*V*-reductions. Assume that the reduced kinetic system satisfies the detailed balance property. Then we have two possibilities:

1. either the concentrations $n_U:=( n_i)_{i \in U }$ satisfy (5.2) for a vector of energies $E \in \mathbb {R}^N$ satisfying (3.4);
2. or, alternatively, we have that for every $\delta >0$ there exists a map $\mathcal {K}_\delta : \mathcal {R} \rightarrow \mathbb {R}_+$ such that
  $$ \Vert \mathcal {K}- \mathcal {K}_\delta \Vert < \delta $$
  and such that $(\Omega , \mathcal {R}, \overline{\mathcal {K}}_\delta [n_U])$ does not satisfy the detailed balance condition.

#### Proof

Assume that the detailed balance property of the reduced system holds. Let $c \in \mathcal {C}_{V } \setminus p(\mathcal {C})$. Then we have that

5.4 $$\begin{aligned} \prod _{j=1}^{|\mathcal {R}_V|/2} \left( \frac{K_{\overline{R}_j} [n_U]}{K_{-\overline{R}_j} [n_U]} \right) ^{c(j)} =1 , \end{aligned}$$

where $\{ \overline{R}_j\}_{j=1}^{|\mathcal {R}_V|/2} = \mathcal {R}_V$. Since we are assuming that all the reactions in the cycles in $\mathcal {C}_V$ are one-to-one, the definition of $\mathcal {K}[n_U]$ together with (5.4) implies that

$$\begin{aligned} \sum _{j=1}^{|\mathcal {R}_V|/2} c(j) \log \left( \frac{K_{R_j}}{K_{-R_j}} \right) = \sum _{j=1}^{|\mathcal {R}_V|/2} \sum _{s \in U } c(j) R_j(s) \log (n_s), \end{aligned}$$

where $R_j =\pi _V^{-1} (\overline{R}_j)$. Since the detailed balance property holds we have that $\log \left( \frac{K_R}{K_{-R} }\right) = - \sum _{i \in \Omega } R(i) E(i)$ where *E* is any solution to (3.4). Hence using the fact that $c \in \mathcal {C}_V$ we deduce that

$$\begin{aligned} \sum _{j=1}^{|\mathcal {R}_V|/2} c(j) \log \left( \frac{K_{R_j}}{K_{-R_j}} \right) = - \sum _{j=1}^{|\mathcal {R}_V|/2} c(j) \sum _{i \in \Omega } R_j (i) E(i) = - \sum _{j=1}^{|\mathcal {R}_V|/2} c(j) \sum _{i \in U } R_j (i) E(i). \end{aligned}$$

We deduce that the detailed balance property of the reduced system holds if and only if for every $c \in \mathcal {C}_V$ we have that

5.5 $$\begin{aligned}&- \sum _{j=1}^{|\mathcal {R}_V|/2} c(j) \sum _{i \in U \cap \mathcal {D}(\mathcal {C}_V) } R_j (i) E(i) = - \sum _{j=1}^{|\mathcal {R}_V|/2} c(j) \sum _{i \in U } R_j (i) E(i)\nonumber \\&= \sum _{j=1}^{|\mathcal {R}_V|/2} \sum _{i \in U } c(j) R_j(i) \log (n_i) \nonumber \\&= \sum _{j=1}^{|\mathcal {R}_V|/2} \sum _{i \in U \cap \mathcal {D}(\mathcal {C}_V) } c(j) R_j(i) \log (n_i). \end{aligned}$$

Notice that since $$c \in \mathcal {C}_V \setminus p(\mathcal {C}) $$ we have that $$ \sum _{j=1}^{|\mathcal {R}_V|/2} c(j) R_j(\ell ) \ne 0 $$ for at least an $$\ell \in U \cap \mathcal {D}(\mathcal {C}_V)$$. Assume that $$n_U $$ has not the form (5.2), we want to prove that it is possible to construct a perturbation of the rate function $$\mathcal {K}_\delta $$ that is such that (5.5) fails for these rates, hence the kinetic system $$(V, \mathcal {R}_V, \mathcal {K}_\delta [n_U]) $$ does not satisfy the detailed balance property. Then we can define the perturbed rate function $$\mathcal {K}_\delta : \mathcal {R} \rightarrow \mathbb {R}_+ $$ as $$\mathcal {K}_\delta (R)=\mathcal {K} (R)$$ for every $$R \in \mathcal {R}_s $$ and

$$ \mathcal {K}_\delta (- R) =\mathcal {K}_\delta ( R) e^{\sum _{i \in \Omega } R(i) (E(i)+ E_\delta (i))} =\mathcal {K}(-R) e^{\sum _{i \in \Omega } R(i) E_\delta (i)} $$

for every $R \in \mathcal {R}_s$. Here we have that $E_\delta (i)=0$ for every $i \ne \ell$ and $E_\delta (\ell ) < \frac{\delta }{\max _{R \in \mathcal {R}_s} |R(\ell )|\max _{ R \in \mathcal {R} } \mathcal {K}(R)}$. By construction we have that $E+E_\delta$ is an energy of the perturbed kinetic system $(\Omega , \mathcal {R}, \mathcal {K}_\delta )$ and the perturbed rates $\mathcal {K}_\delta$ satisfy the assumptions of the theorem indeed we have that by construction $(\Omega , \mathcal {R}, \mathcal {K}_\delta )$ satisfies the detailed balance property and moreover we have that

$$\begin{aligned} &  \Vert \mathcal {K} - \mathcal {K}_\delta \Vert \le \max _{ R \in \mathcal {R} } |\mathcal {K}(R)- \mathcal {K}_\delta (R)| \le \max _{R\in \mathcal {R}} \mathcal {K} (R) \max _{ R \in \mathcal {R} } |1 - e^{E_\delta (\ell ) R(\ell ) } | \\ &  \quad \le \max _{ R \in \mathcal {R} } \mathcal {K}(R) \max _{ R \in \mathcal {R} } |E_\delta (\ell ) R(\ell ) | < \delta . \end{aligned}$$

In order to obtain the above inequalities we are using the fact that for sufficiently small $$x >0$$ we have that $$1- e^{x } < x $$. Moreover, equality (5.5) fails for the kinetic system $$(\Omega , \mathcal {R}, \mathcal {K}_\delta )$$. Indeed we have that

$$\begin{aligned}&\sum _{j=1}^{v/2} c(j) \sum _{i \in U \cap \mathcal {D}(\mathcal {C}_V) } R_j (i) [E(i)+ E_\delta (i) ] + \sum _{j=1}^{v/2} \sum _{i \in U \cap \mathcal {D}(\mathcal {C}_V) } c(j) R_j(i) \log (n_i)\\&\quad = E_\delta (\ell ) \sum _{j=1}^{v/2} c(j) R_j (\ell ) \ne 0 \end{aligned}$$

that, therefore, the kinetic system $(\Omega , \mathcal {R}, \mathcal {K}_\delta [n_U] )$ does not satisfy the detailed balance property. $\square$

In the following example, we show that the assumption that the reactions in $\mathcal {C}_V$ are 1-1 reactions is needed for Theorem 5.4 to hold.

#### Example 5.7

Let $(\Omega , \mathcal {R})$ be the network in Example 4.3. As already anticipated in Example 4.6, the space of the cycles of this chemical network is

$$ \mathcal {C} = \operatorname {span} \{ c_1, c_2 \} $$

with $c_1=(1,0,1,-1,0,1)$ and $c_2 =(0,1,1,-1,1,0)$. Consider the *U* reduced kinetic system with $U=\{5,6\}$. Then we obtain that

$$ \mathcal {C}_{V} = \operatorname {span}(c) $$

where $$c=(1,1,-1,1)$$. Notice that $$p[c_1] = p[c_2] = c $$. As a consequence in this example it holds that $$p (\mathcal {C}) = \mathcal {C}_{V } $$. Assume that

$$\begin{aligned} &  K_{R_1} = K_{-R_1} = K_{R_2} =K_{-R_2} = K_{R_3}= K_{-R_3} = K_{R_4} \\ &  \quad = K_{-R_4} = K_{-R_5} = K_{R_5} =K_{R_6} =K_{-R_6} =1. \end{aligned}$$

Clearly for every cycle $c \in \mathcal {C}$ we have that

$$ \prod _{j=1}^{6} \left( \frac{ K_{R_j} }{K_{-R_j}} \right) ^{c(j) } =1. $$

As a consequence the non reduced kinetic system $(\Omega , \mathcal {R}, \mathcal {K} )$ satisfies the detailed balance property. Let $\tilde{R}_i$, for $i=1,2,3,4$ be the reactions of the reduced system computed in Example 4.3. Equality (4.5) implies that the rates of the reduced kinetic system are given by

$$ K_{\tilde{R}_4}[n_U]= K_{R_5} + K_{R_6} = 2 \ \text { and } \ K_{-\tilde{R}_4}[n_U] = K_{-R_{6}} n_5 +K_{-R_{5}} n_6 = n_5 + n_6, $$

similarly we have that

$$ K_{\tilde{R}_1}[n_U]= n_5+n_6 \ \text { and } \ K_{-\tilde{R}_1}[n_U] =2. $$

In this case it is easy to see that for every cycle $c \in \mathcal {C}_V$ we have

$$ \prod _{j=1}^{4} \left( \frac{ K_{\tilde{R}_j} [n_U]}{K_{-\tilde{R}_j} [n_U]} \right) ^{c(j) } =1. $$

Hence the reduced kinetic system satisfies the detailed balance property. Assume instead that

$$ K_{R_1} = K_{-R_1} = K_{R_2} =K_{-R_2} = K_{R_3}= K_{-R_3} = K_{R_4} = K_{-R_4} = K_{-R_5} = K_{R_5} =1. $$

while

$$ K_{R_6} = 2, \ K_{-R_6} = 2. $$

Notice that by construction for every cycle $c \in \mathcal {C}$ we have that

$$ \prod _{j=1}^{6} \left( \frac{ K_{R_j} }{K_{-R_j}} \right) ^{c(j) } =1. $$

We conclude that $(\Omega , \mathcal {R}, \mathcal {K})$ satisfies the detailed balance property. However, in this case we have that

$$ K_{\tilde{R}_4}[n_U]= K_{R_5} + K_{R_6} = 3 \ \text { and } \ K_{-\tilde{R}_4}[n_U] = ( K_{-R_{6}} n_5 +K_{-R_{5}} n_6) = n_6 + 2 n_5 $$

while $K_{\tilde{R}_1}[n_U]= n_5+n_6$ while $K_{-\tilde{R}_1}[n_U] =2$. Therefore for $c=(1,1,-1,1)$ we have that

$$ \prod _{j=1}^{4} \left( \frac{ K_{R_j}[n_U] }{K_{-R_j} [n_U]} \right) ^{c(j) } = \frac{3 (n_5+n_6) }{2(n_6+ 2n_5) } \ne 1 $$

for $$n_5 \ne n_6 $$. Hence, the detailed balance property in this case holds only if the concentrations of $$n_5, n_6 $$ are chosen at equilibrium values, i.e. if they are such that $$n_5=N_5$$ and $$n_6=N_6$$ where *N* is the steady state of the kinetic system $$(\Omega , \mathcal {R}, \mathcal {K} ) $$.

The example above motivates the following proposition.

#### Proposition 5.8

Assume that the kinetic system $(\Omega , \mathcal {R}, \mathcal {K} )$ satisfies the detailed balance property. Let *U* and *V* be as in Definition 4.1. Assume moreover that there exists at least one reaction $R \in \mathcal {C}_V$ that is not a 1-1 reaction. Assume that the reduced system $(V, \mathcal {R}_V, \mathcal {K} [n_U])$ satisfies the detailed balance. Then we have two possibilities:

1. either the concentrations $n_U:=\{ n_i\}_{i \in U }$ satisfy (5.2);
2. or, alternatively, we have that for every $\delta >0$ there exists a map $\mathcal {K}_\delta : \mathcal {R} \rightarrow \mathbb {R}_+$ such that
  $$ \Vert \mathcal {K}- \mathcal {K}_\delta \Vert < \delta $$
  and such that $(\Omega , \mathcal {R}, {\mathcal {K}}_\delta [n_U])$ does not satisfy the detailed balance condition.

#### Proof

Assume that the kinetic system $(V, \mathcal {R}_V, \mathcal {K}[n_U] )$ satisfies the detailed balance property. Then we must have that if $c \in \mathcal {C}_V$, then

$$ \sum _{j=1}^{|\mathcal {R}_V|/2} c(j) \mathcal {E}(\overline{R}_j)=0 $$

where $\{ \overline{R}_j\}_{j=1}^v =\mathcal {R}_V$. Assume that $c \in \mathcal {C}_V$ contains at least a reaction that is not one to one. Notice that for every $j \in \{1, \dots , |\mathcal {R}_V|/2\}$ we have that

$$\begin{aligned} \mathcal {E}(\overline{R}_j)=\log \left( \frac{\sum _{R_{k_j} \in \pi _V^{-1} (\overline{R}_j)} \hat{K}_{- R_{k_j}}}{\sum _{R_{k_j} \in \pi _V^{-1} (\overline{R}_j) } \hat{K}_{ R_{k_j}}}\right) \end{aligned}$$

where $\hat{K}_{R_{k_j}} = K_{R_{k_j}} \prod _{i \in U \cap I(R_j)} n_i^{-R_{k_j}(i)}$ and $\hat{K}_{-R_{k_j}} = K_{-R_{k_j}} \prod _{i \in U \cap F(R_{k_j})} n_i^{R_{k_j}(i)}$. The detailed balance property of $(\Omega , \mathcal {R}, \mathcal {K} )$ implies that $K_{-R_{k_j}} = K_{R_{k_j}} e^{\sum _{i \in \Omega }R_{k_j}(i) E(i)}$ for every *E* solution to (3.4). Hence

$$ \frac{\hat{K}_{-R_{k_j}}}{\hat{K}_{R_{k_j}}} = e^{\sum _{i \in \Omega }R_{k_j}(i) E(i)} \prod _{i \in U } n_i^{R_{k_j}(i)}. $$

We deduce that

$$\begin{aligned} \mathcal {E}(\overline{R}_j)&=\log \left( \frac{\sum _{R_{k_j} \in \pi _V^{-1} (\overline{R}_j)} \hat{K}_{ R_{k_j}}e^{\sum _{i \in \Omega }R_{k_j}(i) E(i)} \prod _{i \in U } n_i^{R_{k_j}(i)}}{\sum _{R_{k_j}\in \pi _V^{-1} (\overline{R}_j) } \hat{K}_{R_{k_j}}}\right) \\&= \sum _{k \in V} \overline{R}_j(k) E(k) +\log \left( \frac{\sum _{R_{k_j} \in \pi _V^{-1} (\overline{R}_j)} \hat{K}_{ R_{k_j}} \prod _{i \in U } (n_ie^{E(i)})^{R_{k_j}(i)}}{\sum _{R_{k_j}\in \pi _V^{-1} (\overline{R}_j) } \hat{K}_{R_{k_j}}}\right) . \end{aligned}$$

The detailed balance property of the reduced system implies that

$$\begin{aligned} 0= \sum _{j=1}^{|\mathcal {R}_V|/2} c(j) \mathcal {E}(\overline{R}_j)&= \sum _{k \in V} E(k) \sum _{j=1}^{|\mathcal {R}_V|/2} c(j)\overline{R}_j(k) + \sum _{j=1}^{|\mathcal {R}_V|/2} c(j) \lambda _j= \sum _{j=1}^{|\mathcal {R}_V|/2} c(j) \lambda _j, \end{aligned}$$

where

$$ \lambda _j:= \log \left( \frac{\sum _{R_{k_j} \in \pi _V^{-1} (\overline{R}_j)} \hat{K}_{ R_{k_j}} \prod _{i \in U } (n_ie^{E(i)})^{R_{k_j}(i)}}{\sum _{R_{k_j} \in \pi _V^{-1} (\overline{R}_j) } \hat{K}_{ R_{k_j}}}\right) $$

We have two options, either $$n_U $$ is given by (5.2), or alternatively,

$$ \prod _{i \in U } (n_ie^{E(i)})^{R_{k_j}(i)} = \alpha _{k_j} \ne 1 \text { for some } j \in \{1, \dots , |\mathcal {R}_V|/2\} $$

and

$$ \sum _{j=1}^{|\mathcal {R}_V|/2} c(j) \log \left( \frac{\sum _{R_{k_j} \in \pi _V^{-1} (\overline{R}_j)} \hat{K}_{ R_{k_j}} \alpha _{k_j} }{\sum _{R_{k_j} \in \pi _V^{-1} (\overline{R}_j) } \hat{K}_{ R_{k_j}}}\right) =0. $$

Without loss of generality we can assume that $\ell \in \{ 1, \dots , |\mathcal {R}_V|/2\}$ is such that $c(\ell ) \ne 0$ and $\alpha _{k_\ell } \ne 1$. Consider a reaction $R_{\overline{k}_\ell } \in \pi ^{-1}_V (R_\ell )$, and define the rate function ${\mathcal {K}}_\delta : \mathcal {R} \rightarrow \mathbb {R}_*$ such that

$$\begin{aligned} &  {\mathcal {K}}_\delta (R)=\mathcal {K} (R) \text { for every } R \ne R_{\overline{k}_\ell }, - R_{\overline{k}_\ell } \text { and } {\mathcal {K}}_\delta ( R_{\overline{k}_\ell })=\mathcal {K} (R_{\overline{k}_\ell }) + \delta ,\\ &  \quad {\mathcal {K}}_\delta (- R_{\overline{k}_\ell })=\mathcal {K}_\delta (R_{\overline{k}_\ell }) e^{ \sum _{ i \in \Omega } R_{\overline{k}_\ell }(i) E(i) } \end{aligned}$$

for $\delta >0$. By construction we have that the kinetic system $(\Omega , \mathcal {R}, \mathcal {K}_\delta )$ satisfies the detailed balance property and that $\Vert \mathcal {K} - \mathcal {K}_\delta \Vert < \delta$. Notice also that the energies of the unperturbed kinetic system $(\Omega , \mathcal {R}, \mathcal {K})$ are also energies for $(\Omega , \mathcal {R}, \mathcal {K}_\delta )$. We denote with $\hat{K}^{(\delta )}_{ R_j}$ the rates induced by $\mathcal {K}_\delta$ via (4.6). Then

$$\begin{aligned}&\sum _{j=1}^{|\mathcal {R}_V|/2} c(j) \log \left( \frac{\sum _{R_{k_j }\in \pi _V^{-1} (\overline{R}_j)} \hat{K}^{(\delta )}_{ R_{k_j }} \alpha _{k_j } }{\sum _{R_{k_j } \in \pi _V^{-1} (\overline{R}_j) } \hat{K}^{(\delta )}_{ R_{k_j }}}\right) \\&= \sum _{j=1, j \ne \ell }^{|\mathcal {R}_V|/2} c(j) \log \left( \frac{\sum _{R_{k_j } \in \pi _V^{-1} (\overline{R}_j)} \hat{K}_{ R_{k_j }} \alpha _{k_j } }{\sum _{R_{k_j } \in \pi _V^{-1} (\overline{R}_j) } \hat{K}_{ R_{k_j }}}\right) \\&+ c(\ell ) \log \left( \frac{\sum _{ R_{k_\ell } \in \pi _V^{-1} (\overline{R}_\ell )} \hat{K}^{(\delta )}_{ R_{ k_\ell }} \alpha _{k_\ell } }{\sum _{R_{k_\ell } \in \pi _V^{-1} (\overline{R}_\ell ) } \hat{K}^{(\delta )}_{ R_\ell }}\right) . \end{aligned}$$

We now take the derivative in $\delta$ and obtain that

$$\begin{aligned} \frac{d}{d\delta } \sum _{j=1}^{|\mathcal {R}_V|/2} c(j) \log \left( \frac{\sum _{R_{k_j }\in \pi _V^{-1} (\overline{R}_j)} \hat{K}^{(\delta )}_{ R_{k_j }} \alpha _{k_j } }{\sum _{R_{k_j } \in \pi _V^{-1} (\overline{R}_j) } \hat{K}^{(\delta )}_{ R_{k_j }}}\right) = c(\ell ) \alpha _{\overline{k}_\ell }&\ne 0. \end{aligned}$$

Since we have that $\sum _{j=1}^{|\mathcal {R}_V|/2} c(j) \left( \frac{\sum _{R_{k_j }\in \pi _V^{-1} (\overline{R}_j)} \hat{K}^{(0)}_{ R_{k_j }} \alpha _{k_j } }{\sum _{R_{k_j } \in \pi _V^{-1} (\overline{R}_j) } \hat{K}^{(0)}_{ R_{k_j }}}\right) =0$, this implies that

$$ \sum _{j=1}^{|\mathcal {R}_V|/2} c(j) \left( \frac{\sum _{R_{k_j }\in \pi _V^{-1} (\overline{R}_j)} \hat{K}^{(\delta )}_{ R_{k_j }} \alpha _{k_j } }{\sum _{R_{k_j } \in \pi _V^{-1} (\overline{R}_j) } \hat{K}^{(\delta )}_{ R_{k_j }}}\right) \ne 0. $$

Therefore $(\Omega , \mathcal {R}_V,{\mathcal {K}}_\delta [n_U])$ does not satisfy the detailed balance property. $\square$

In the following example we show that also the assumption that the reactions in the cycles of the non-reduced kinetic system have non zero projection in the reduced system is needed in Theorem 5.4.

#### Example 5.9

Let $(\Omega , \mathcal {R})$ be the network in Example 4.3 and Example 5.7. If

$$\begin{aligned} &  K_{R_1} = K_{-R_1} = K_{R_2} =K_{-R_2} = K_{R_3}= K_{-R_3} = K_{R_4} \\ &  \quad = K_{-R_4} = K_{-R_5} = K_{R_5} =K_{R_6} =K_{-R_6} =1, \end{aligned}$$

then $(\Omega , \mathcal {R})$ satisfies the detailed balance property. Consider the reduced system induced by $\overline{U}=\{ 1, 4\}$. Notice that, as shown in Example 4.3, we have that $\pi _{\overline{V}} R_4=0$. As a consequence Assumption 2. in Theorem 5.4 fails. Let $\tilde{R}_i$, for $i=1,2,3,4,5$ be the reactions of the reduced system computed in Example 4.3. Equality (4.5) implies that

$$ K_{\tilde{R}_1}[n_U]= n_1,\ K_{\tilde{R}_2}[n_U]= n_1, \ K_{-\tilde{R}_4}[n_U] =n_4, \ K_{-\tilde{R}_5}[n_U] =n_4 $$

while the rest of the rates are equal to 1. A cycle of the reduced system is $c=(1,1,2,1,1)$. Notice that

$$ \prod _{j=1}^5 \left( \frac{K_{- \tilde{R}_j}}{K_{\tilde{R}_j}} \right) ^{c(j)} =1 $$

is true only when $n_1=n_4$. This means that the detailed balance property of the reduced system holds only if $n_1=n_4$.

Motivated by the example above we prove the following statement.

#### Proposition 5.10

Assume that the kinetic system $(\Omega , \mathcal {R}, \mathcal {K} )$ satisfies the detailed balance property. Let *U* and *V* be as in Definition 4.1. Assume moreover that every reaction $R \in \mathcal {C}_V$ is a 1-1 reaction. Moreover, we assume that there exists at least a cycle $c_V \in \mathcal {C}_V$ that is such that every $c \in \mathcal {C}$ satisfying $p[c]= c_V$ contains at least one reaction with zero-*V*-reduction. Assume that the reduced system $(V, \mathcal {R}_V, \mathcal {K} [n_U])$ satisfies the detailed balance. Then we have to possibilities:

1. either the concentrations $n_U:=\{ n_i\}_{i \in U }$ satisfy (5.2);
2. or, alternatively, we have that for every $\delta >0$ there exists a map $\mathcal {K}_\delta : \mathcal {R} \rightarrow \mathbb {R}_+$ such that
  $$ \Vert \mathcal {K}- \mathcal {K}_\delta \Vert < \delta $$
  and such that $(\Omega , \mathcal {R}, {\mathcal {K}}_\delta [n_U])$ does not satisfy the detailed balance condition.

#### Proof

By assumption we know that there exist a reactions in $\mathcal {C}$ with zero-*V*-reduction. Therefore, there exists a $k \in \{ 1, \dots , r /2\}$ such that the reaction $R_k = {\textbf {R}} e_k$ is such that $R_k \in c$ (i.e. $c(k) \ne 0$) for $c \in \mathcal {C}$ and $\pi _V R_k=0$. As a consequence, the detailed balance property of the kinetic system $(\Omega , \mathcal {R}, \mathcal {K})$ implies that for every $c \in \mathcal {C}$ we have that

$$\begin{aligned} 0&= \sum _{j=1}^{|\mathcal {R}|/2} c(j) \mathcal {E}(R_j) = \sum _{ \{ j: \pi _V R_j =0\} } c(j) \mathcal {E}(R_j) + \sum _{ \{ j: \pi _V R_j \ne 0 \} } c(j) \mathcal {E}(R_j) \\&= \sum _{ \{ j: \pi _V R_j = 0 \} } c(j) \sum _{i \in U } E(i) R_j(i) + \sum _{ \{ j: \pi _V R_j \ne 0 \} } c(j) \mathcal {E}(R_j). \end{aligned}$$

As a consequence we have

5.6 $$\begin{aligned} \sum _{ \{ j: \pi _V R_j = 0 \} } c(j) \sum _{i \in U } E(i) R_j(i) =- \sum _{ \{ j: \pi _V R_j \ne 0 \} } c(j) \mathcal {E}(R_j). \end{aligned}$$

Since the reactions in the cycle $$\mathcal {C}_V $$ are 1-1 we have that $$p[c] \in \mathbb {R}^{|\{ i \in \{ 1, \dots , r/2 \}: \pi _V R_i \ne 0 \} | } $$ and $$p[c] (i) = c (i) $$ for every *i* such that $$\pi _V R_i \ne 0.$$ Recall that $$p[c] \in \mathcal {C}_V $$ and recall that by assumption we know that $$p[c] \ne 0$$. As a consequence if we assume that the kinetic system $$(V, \mathcal {R}_V, \mathcal {K} [n_U]) $$ satisfies the detailed balance condition, then we must have that

$$ \sum _{ \{ j: \pi _V R_j \ne 0 \} } c(j) \mathcal {E}(\pi _V R_j)=0. $$

This implies that

$$\begin{aligned} 0=\sum _{ \{ j: \pi _V R_j \ne 0 \} } c(j) \mathcal {E}(\pi _V R_j)= \sum _{ \{ j: \pi _V R_j \ne 0 \} } c(j) \mathcal {E}(R_j) - \sum _{ \{ j: \pi _V R_j \ne 0 \} } c(j)\sum _{i \in U} \log (n_i) R_j(i). \end{aligned}$$

Hence we have that

$$ \sum _{ \{ j: \pi _V R_j \ne 0 \} } c(j) \mathcal {E}(R_j) = \sum _{ \{ j: \pi _V R_j \ne 0 \} } c(j)\sum _{i \in U} \log (n_i) R_j(i). $$

This together with equality (5.6) implies that

$$\begin{aligned}&\sum _{ \{ j: \pi _V R_j \ne 0 \} } c(j)\sum _{i \in U} \log (n_i) R_j(i) = \sum _{ \{ j: \pi _V R_j \ne 0 \} } c(j) \mathcal {E}(R_j) = - \sum _{ \{ j: \pi _V R_j = 0 \} } c(j) \mathcal {E}(R_j) \\&\quad = - \sum _{ \{ j: \pi _V R_j = 0 \} } c(j) \sum _{i \in \Omega } E(i) R_j(i) = - \sum _{ \{ j: \pi _V R_j = 0 \} } c(j) \sum _{i \in U } E(i) R_j(i). \end{aligned}$$

As a consequence in order to have that the reduced kinetic system satisfies the detailed balance property we need to have

5.7 $$\begin{aligned} &  \sum _{ \{ j: \pi _V R_j \ne 0 \} } c(j)\sum _{i \in U} \log (n_i) R_j(i) + \sum _{ \{ j: \pi _V R_j = 0 \} } c(j) \sum _{i \in U } E(i) R_j(i)=0. \end{aligned}$$

Assume that $n_i$ is not given by (5.2). Then we can construct a perturbed kinetic system that is such that $(V, \mathcal {R}_V, \mathcal {K}_\delta )$ satisfies the detailed balance property but the reduced system does not satisfy (5.7) and hence does not satisfy the detailed balance property. To this end we can argue exactly as in the proof of Proposition 5.6. Indeed, we can define the perturbed rate function $\mathcal {K}_\delta : \mathcal {R} \rightarrow \mathbb {R}_+$ as $\mathcal {K}_\delta (R)=\mathcal {K} (R)$ for every $R \in \mathcal {R}_s$ and

$$ \mathcal {K}_\delta (- R) =\mathcal {K}_\delta ( R) e^{\sum _{i \in \Omega } R(i) (E(i)+ E_\delta (i))} =\mathcal {K}(-R) e^{\sum _{i \in \Omega } R(i) E_\delta (i)} $$

for every $R \in \mathcal {R}_s$. Here we have that $E_\delta (i)=0$ for every $i \ne \ell$ and $E_\delta (\ell ) < \frac{\delta }{\max _{R \in \mathcal {R}_s} |R(\ell )|\max _{ R \in \mathcal {R} } \mathcal {K}(R)}$ for $\ell \in U$ such that

$$ \sum _{ \{ j: \pi _V R_j = 0 \} } c(j) R_j(\ell ) \ne 0. $$

Notice that such an $\ell \in U$ exists. Indeed assume by contradiction that for every $i \in U$

$$ \sum _{ \{ j: \pi _V R_j = 0 \} } c(j) R_j(i) = 0. $$

This implies that $\sum _{ \{ j: \pi _V R_j \ne 0 \} } c(j) R_j(i) = 0$. Hence (upon reordering the reactions), the vector $\tilde{c}\in \mathbb {R}^{r/2}$ defined as $\tilde{c}(i)=c(i)$ for every *i* such that $\pi _V R_i \ne 0$ and such that $\tilde{c} (i)=0$ otherwise is a cycle, i.e. it belongs to $\mathcal {C}$ and is such that $p[\tilde{c}]=c$. This contradicts the assumption of the Proposition.

As in Proposition 5.6 we prove that the rate function $\mathcal {K}_\delta$ satisfies the assumptions of the Proposition. Moreover, this new rate function does not satisfy (5.7) with respect to the perturbed energy $\overline{E}= E+ E_\delta$ indeed

$$\begin{aligned}&\sum _{ \{ j: \pi _V R_j \ne 0 \} } c(j)\sum _{i \in U} \log (n_i) R_j(i) + \sum _{ \{ j: \pi _V R_j = 0 \} } c(j) \sum _{i \in U } (E(i)\\&\quad + E_\delta (i) ) R_j(i)= E_\delta (\ell ) \sum _{ \{ j: \pi _V R_j = 0 \} } c(j) R_j(\ell ) . \end{aligned}$$

Now recall that $\sum _{ \{ j: \pi _V R_j = 0 \} } c(j) R_j(\ell ) \ne 0$. $\square$

We conclude by summarizing the results obtained in this section. In Theorem 5.4 we state some sufficient conditions that guarantee that if the non reduced kinetic system satisfies the detailed balance property, then also the reduced kinetic system satisfies the detailed balance property in a stable manner. In Proposition 5.8 we have proven that the fact that the cycles of the reduced system contain only reactions that are 1-1 is a necessary condition for the stability of the detailed balance property of the reduced kinetic system. However this property is not sufficient to have that the reduced kinetic system satisfies the detailed balance property in a stable way. This is shown by Example 5.9.

In Proposition 5.10, we find another necessary condition for the stability of the detailed balance property in the reduced system. This necessary condition is the following. If a cycle $c_V$ of the reduced system can be written as the projection *p*[*c*] of a cycle *c* of the non reduced kinetic system, then *c* does not contain reactions that project to zero. Finally in Proposition 5.6 we prove that the fact that the projection of the cycles of the non reduced system is equal to the set of the cycles of the reduced system is also a necessary condition for the stability of the detailed balance property. Summarizing in this section we find three necessary and sufficient conditions conditions to obtain a reduced kinetic system that satisfies the detailed balance property in a stable manner (i.e. stable under small perturbation of the chemical rates or of the frozen concentrations).

## Completion of a Kinetic System

As already mentioned in the previous sections, every kinetic system that does not exchange matter with the environment must satisfy the detailed balance property. However, as explained in the previous section, kinetic systems that do not satisfy the detailed balance property can be obtained as a result of the reduction of a kinetic system with detailed balance. In this section we characterize the kinetic systems that do not satisfy the detailed balance condition and that are obtained by the reduction of closed kinetic system. In particular, as expected, in Theorem 6.3 we prove that every kinetic system that is not closed can be obtained as the reduction of a closed kinetic system. Notice that in the literature there are many examples of chemical systems that are not closed, see for instance the models of adaptation in Ferrell (2016).

We then consider the case in which some reactions of the open kinetic system cannot be modified. This is the case when it is possible to establish experimentally that some chemical reactions are exactly as they appear in the open kinetic system and the substances that take part to these chemical reactions belong to the set of substances of the open kinetic system. In this case we prove that it is possible to obtain this open kinetic system as a reduction of a larger closed kinetic system (its completion) if the reactions that cannot be modified are not part of the cycles of the reduced system. We also prove that if all the reactions in the cycles of the kinetic system cannot be modified then the kinetic system does not admit a closed completion. However, we stress that we do not prove that an open kinetic system admits a closed completion if and only if all the reactions in its cycles can be modified. Indeed as shown in Example 6.9, in some cases, it is enough to be able to modify one single reaction for each cycle to be able to construct a closed completion of the open kinetic system.

### Definition 6.1

*(Completion of a chemical network and of a kinetic system)* Let $(\Omega , \mathcal {R})$ be a bidirectional chemical network. A completion of $(\Omega , \mathcal {R})$ is a chemical network $(\Omega _c, \mathcal {R}_c)$ such that

1. $$\Omega \subset \Omega _c$$;
2. $$\mathcal {R}= \{ \pi _{\Omega } R: R \in \mathcal {R}_c \} $$.

A completion of a kinetic system $(\Omega , \mathcal {R}, \mathcal {K})$ is a kinetic system $(\Omega _c, \mathcal {R}_c, \mathcal {K}_c)$ that is such that $(\Omega _c, \mathcal {R}_c)$ is a completion of the chemical network $(\Omega , \mathcal {R})$ and there exists a set of concentrations $n_{\Omega _c \setminus \Omega }:={(n_i)}_{i \in \Omega _c \setminus \Omega }$, where $\mathcal {K} = \mathcal {K}_c [n_{\Omega _c \setminus \Omega }]$.

### Completion Without Constraints

In this section we prove that every open kinetic system $(\Omega , \mathcal {R}, \mathcal {K})$ admits a closed completion. To this end we first prove that the chemical network $(\Omega , \mathcal {R})$ admits a completion $(\Omega _c, \mathcal {R}_c)$ that is conservative, that is such that every reaction satisfies (3.11) and that does not have cycles. Notice that this implies that for every choice of rate function $\mathcal {K}_c$ we have that $(\Omega _c, \mathcal {R}_c, \mathcal {K}_c)$ is closed. Hence every kinetic system $(\Omega , \mathcal {R}, \mathcal {K})$ admits a closed completion.

#### Proposition 6.2

Assume that $(\Omega , \mathcal {R})$ is a bidirectional chemical network. Then there exists a conservative completion $(\Omega _c, \mathcal {R}_c)$ of $(\Omega , \mathcal {R})$. Moreover this completion satisfies the following conditions

- every reaction $R \in \mathcal {R}_c$ satisfies (3.11),
- $\mathcal {C}_c = \{ 0\}$, i.e. the completion does not have cycles,
- every $R \in \mathcal {R}_c$ is a reaction with non zero-$\Omega$-reduction and every $R \in \mathcal {R}$ is a 1-1 reduction.

#### Proof

The proof of this proposition is structured as follows. As a first step we consider a chemical network that has sources and sinks, hence that does not satisfy (3.11). We explain how to complete this system by adding new substances and to obtain a completion $(\Omega _q, \mathcal {R}_q)$ whose reactions satisfy (3.11). As a second step we assume that $(\Omega _q, \mathcal {R}_q )$ is non-conservative and show how to complete it to obtain a completion $(\Omega _e, \mathcal {R}_e)$ that is conservative. Moreover the way in which the completion is constructed guarantees that no sources and sinks are produced in the completion, hence $(\Omega _e, \mathcal {R}_e)$ does not have sources and sinks. As a third step we show how to complete the chemical network $(\Omega _e, \mathcal {R}_e)$ to obtain a chemical network $(\Omega _c, \mathcal {R}_c)$ that has no cycles. Also in this case we construct the completion in such a way that no sources and sinks are produced and in such a way that $(\Omega _c, \mathcal {R}_c)$ is conservative.

**Step 1: Constructing a completed kinetic system that does not have sources and sinks.** Assume that $(\Omega , \mathcal {R})$ is such that the set $Q \subset \mathcal {R}$ defined as

$$ {Q:= \{ R \in \mathcal {R} : I(R) = \emptyset \} \cup \{ R \in \mathcal {R} : F(R)=\emptyset \}} $$

is not empty. Let us define $q:=|Q|$. Without loss of generality we consider a reaction $\overline{R} \in Q$ such that either $I(\overline{R})=0$ or $F(\overline{R})=0$. We define the extended set of substances $\Omega _1:=\Omega \cup \{N+1, N+2 \}$. The set of extended reactions is defined as

$$ \mathcal {R}_1:= \left\{ \left( \begin{array}{c} R \\ 0 \\ 0 \end{array} \right) : R \in \mathcal {R} \quad R \ne \overline{R}, \ -R \ne \overline{R} \right\} \cup \left( \begin{array}{c} \overline{R} \\ \alpha \\ -\alpha \end{array} \right) \cup \left( \begin{array}{c} -\overline{R} \\ - \alpha \\ \alpha \end{array} \right) $$

for some $\alpha \ne 0$. Notice that by construction we have that the reaction $\overline{R}_q:= (\overline{R}, \alpha , - \alpha )^T$ is such that $\pi _\Omega \overline{R}_q = \overline{R}$ and is such that $I(\overline{R}_q) \ne 0$ and $F(\overline{R}_q) \ne 0$. We can iterate the procedure adding two substances to the network for every reaction $R \in \mathcal {R}$ that is such that either $I(R)=\emptyset$ or $F(R)=\emptyset$. We obtain a chemical network $(\Omega _q, \mathcal {R}_q)$ that does not contain sources and sinks. Notice that we have that $\Omega _q= \Omega \cup \{ N+1, N+2q\}$. We stress that for every $R \in \mathcal {R}_q$ we have that $\pi _{\Omega } R \ne 0$. Moreover if $R_1, R_2\in \mathcal {R}_q$ are such that $R_1 \ne R_2$, then $\pi _{\Omega } R_1 \ne \pi _{ \Omega } R_2$.

**Step 2: Constructing a conservative network.** Assume that $(\Omega _q, \mathcal {R}_q)$ is non-conservative. We denote with $\mathcal {M}_q$ the set of the conservation laws of $(\Omega _q, \mathcal {R}_q)$. We now explain how to complete it in order to obtain a conservative system. Since $(\Omega _q, \mathcal {R}_q )$ is non-conservative, the set $B \subset \Omega _q$ defined as

$$ B:= \left\{ u \in \Omega : m (u)=0 \ \forall m \in \mathcal {M}_q \right\} $$

is such that $$B \ne \emptyset $$. Let $$ b = |B|$$.

Upon reordering of the elements in $\Omega _q$ we have that $B=\{ N+2q-b +1, \dots , N +2q\}$. We define $A:= \Omega _q \setminus B$. Notice that every $R \in \mathcal {R}_q$ can be written as $$ R=\left( \begin{array}{c} \pi _A R \\ \pi _B R \end{array}\right) $$. We define the set of extended substances as $\Omega _e = \Omega _q \cup \{ N+2q+1, \dots , N+b+2q \}$ and we construct the set of the extended reactions as follows

$$ \mathcal {R}_e := \left\{ R_e = \left( \begin{array}{c} \pi _A R \\ \pi _B R \\ - \pi _B R \end{array}\right) \in \mathbb {R}^{N+2q +b}: R \in \mathcal {R} \right\} . $$

Notice that for every $R \in \mathcal {R}_e$ we have that $\pi _{\Omega _q} R \ne 0$. Moreover if $R_1, R_2\in \mathcal {R}_e$ are such that $R_1 \ne R_2$, then $\pi _{ \Omega _q } R_1 \ne \pi _{ \Omega _q } R_2$. Moreover, each vector $m \in \mathcal {M}_q$ can be written as $$ m= \left( \begin{array}{c} \pi _A m \\ {\textbf {0}}_b \end{array}\right) $$. Let us define the set of vectors

$$ M_e := \left\{ m_e =\left( \begin{array}{c} \pi _A m \\ {\textbf {1}}_{b}\\ {\textbf {1}}_{b} \end{array}\right) \in \mathbb {R}^{N +2q+ b } :m \in \mathcal {M}_q \right\} . $$

Now notice that by definition $M_e \subset \operatorname {span} \{ R_e: R_e \in \mathcal {R}_e \}^{\perp }$. As a consequence, the chemical network $(\Omega _e, \mathcal {R}_e )$, where $\Omega _e= \Omega \cup \{N+2q+1, \dots , N+2q+b \}$ is conservative. Moreover, notice that by construction $(\Omega _e, \mathcal {R}_e )$ satisfies (3.11).

**Step 3: Constructing a completed kinetic system that does not have cycles.** Assume that the chemical network $(\Omega , \mathcal {R} )$ contains some cycles. Then the kinetic system $(\Omega _e, \mathcal {R}_e )$ obtained in Step 1 and Step 2 is conservative, it satisfies (3.11), but it might have cycles. Consider a basis $\{ c_1, \dots , c_d \}$ of the space of the cycles $\mathcal {C}_e$ of the chemical network $(\Omega _e, \mathcal {R}_e )$ and consider a vector $c_i$ of the basis. By definition we have that ${\textbf {R}}_e c_i ={\textbf {0}}$. This implies that there exist *k*, *j* with $k \ne j$ such that $c_i(k) , c_i(j) \ne 0$ and a row $\zeta ^T \in \mathbb {R}^{r/2}$ of the matrix ${\textbf {R}}_e$ such that $\zeta (j) \ne 0$ and $\zeta (k) \ne 0$. Here we used the notation $r=|\mathcal {R}_e|$.

We consider the vector $$W_1 \in \mathbb {R}^{r/2} $$ such that $$W_1(k) = \zeta (k) $$ and $$W_1(\ell ) =0$$ for every $$ \ell \ne k $$. On the other hand we define $$W_2 $$ as $$\zeta - W_1 $$. Notice that by construction $$W_1^T c_i \ne 0 $$ and $$W_2^T c_i \ne 0$$. Therefore the matrix

$$ {\textbf {R}}_c =\left( \begin{array}{c} {\textbf {R}}_e \\ W_1^T \\ W_2^T \\ \end{array} \right) $$

is such that $${\textbf {R}}_c c_i \ne 0$$. The set of reactions $$\mathcal {R}_e $$ induced by the matrix $${\textbf {R}}_e$$ is such that for every $$R \in \mathcal {R}_e $$ we have that $$\pi _{\Omega _e} R \ne 0 $$. Moreover if $$R_1, R_2\in \mathcal {R}_c $$ are such that $$R_1 \ne R_2 $$, then $$\pi _{\Omega _e } R_1 \ne \pi _{\Omega _e} R_2 $$. Therefore the dimension of the space of the cycles of the chemical network $$(\Omega _e \cup \{ N+2q+b, N+2q+b+2\}, \mathcal {R}_c) $$ is 1 less than the dimension of the space of the cycles of $$(\Omega _e, \mathcal {R}_e)$$. Moreover notice that the network that we construct with this procedure is still conservative. Indeed, without loss of generality let us assume that the vector $$\zeta $$ is the *i*-th row of $${\textbf {R}}_e$$, hence $$\zeta = e_i^T {\textbf {R}}_e $$. Since $$(\Omega _e, \mathcal {R}_e) $$ is conservative we have that there exists $$m_e \in \mathcal {M}_e $$ such that $$m_e(i ) \ne 0 $$. Therefore, by construction the vector $$(m_c, \frac{1}{4}m_e(i), \frac{1}{4} m_e(i) )\in \mathbb {R}^{N+ 2 q+b+2 } $$, where $$m_c (j)=m_e(j) $$ for every $$j \ne i $$ and $$m_c(i) =\frac{1}{2} m_e(i)$$ is a conservation law for $$(\Omega _c, \mathcal {R}_c )$$. Finally notice that the kinetic system satisfies (3.11) by construction. Iterating this process we can remove all the cycles and the desired conclusion follows. $$\square $$

#### Theorem 6.3

Assume that $(\Omega , \mathcal {R}, \mathcal {K})$ is a bidirectional kinetic system. Then $(\Omega , \mathcal {R}, \mathcal {K})$ admits a closed completion $(\Omega _c, \mathcal {R}_c, \mathcal {K}_c)$ that is such that every $R \in \mathcal {R}_c$ is a reaction with non zero-$\Omega$-reduction and every $R \in \mathcal {R}$ is a 1-1 reduction.

#### Proof

Proposition 6.2 implies that the chemical network $(\Omega , \mathcal {R})$ admits a completion $(\Omega _c, \mathcal {R}_c)$ that is conservative, that is such that every $R \in \mathcal {R}_c$ satisfy (3.11) and that does not have cycles, hence $\mathcal {C}_c \ne \{ 0\}$. As a consequence for every rate function $\mathcal {K}_c$ we have that $(\Omega _c, \mathcal {R}_c, \mathcal {K}_c)$ is closed, indeed since it has no cycles the circuit condition in Lemma 3.2 holds independently on the reaction rates. In particular the kinetic system $(\Omega _c,\mathcal {R}_c, \mathcal {K})$ is closed and is such that $\mathcal {K}[n_U] = \mathcal {K}$ for $n_{\Omega _c \setminus \Omega } = {\textbf {1}}$. Hence $(\Omega _c,\mathcal {R}_c, \mathcal {K})$ is a closed completion of $(\Omega ,\mathcal {R}, \mathcal {K})$. $\square$

#### Remark 6.4

Notice that to prove Theorem 6.3 we prove that $(\Omega _c,\mathcal {R}_c, \mathcal {K})$ is a closed completion of $(\Omega ,\mathcal {R}, \mathcal {K})$. However we also have the same statement for every rate function $\mathcal {K}_c$ defined as

$$ \mathcal {K}_c(R) = \mathcal {K}(\pi _\Omega R) \prod _{i\in I(R) \cap \Omega _c \setminus \Omega } n_i^{R(i)}, \quad \forall R\in \mathcal {R}_c $$

for any vector $n_{\Omega _c \setminus \Omega }$.

#### Remark 6.5

In Proposition 6.2 we construct a completion for the chemical network $(\Omega , \mathcal {R})$. The completion that we construct does not contain cycles. We then use this completion to prove Theorem 6.3. In particular we use the fact that a kinetic system without cycles satisfies the detailed balance property. However notice that the proof could be optimized. Indeed we could obtain a completion that is closed adding less substances and modifying less reactions. For example, we could construct a completion were we remove only the cycles of the kinetic system $(\Omega , \mathcal {R}, \mathcal {K} )$ on which the circuit condition in Lemma 3.2 does not hold.

### Completion with Constraints

We now consider the case of a kinetic system in which some reactions cannot be modified and study under which conditions it is still possible to obtain a completed kinetic system that is closed. Here when we say that we cannot modify a reaction *R* we mean that the only possible completion $\overline{R}$ of the reaction *R* has the form $$ \left( \begin{array}{c} R \\ {\textbf {0}} \end{array} \right) $$. In other words the completion of *R* involves exactly the same substances as *R*, i.e $D(R )= D(\overline{R})$.

#### Definition 6.6

*(Admissible completions)* Assume that $(\Omega , \mathcal {R})$ is a chemical network. Let $\mathcal {R}_a \subset \mathcal {R}$. We say that a completion $(\Omega _c, \mathcal {R}_c)$ of $(\Omega , \mathcal {R})$ is $\mathcal {R}_a$-admissible if

$$ \pi _{\Omega _c \setminus \Omega } R =0 \quad \forall R \in \mathcal {R}_c \text { s.t. } \pi _{ \Omega } R \in \mathcal {R}_a. $$

We say that $\mathcal {R}_a$ is the set of constrained reactions.

In the following proposition we formulate the assumptions on the set of constrained reactions $\mathcal {R}_a$ that guarantee that a kinetic system admits a closed completion. These conditions are that the constrained reactions do not belong to the cycles of the kinetic system that we want to complete, that the admissible reactions are not sources or sinks and that all the substances that appear in the constrained reactions appear in a conservation law.

#### Proposition 6.7

Assume that $$(\Omega , \mathcal {R}, \mathcal {K}) $$ is a bidirectional kinetic system. Let $$\mathcal {R}_a \subset \mathcal {R}$$ be such that $$\mathcal {R}_a \cap \mathcal {C} = \emptyset $$. Assume that for every $$R \in \mathcal {R}_a $$ we have that $$(3.11) $$ holds. Moreover, assume that for every $$R \in \mathcal {R}_a$$ we have that $$\pi _A R =0 $$ where

$$ {A:= \{ i \in \Omega : m(i)=0, \quad \forall m \in \mathcal {M}\}. } $$

Then, there exists a $\mathcal {R}_a$-admissible closed completion of $(\Omega , \mathcal {R}, \mathcal {K})$.

#### Proof

First of all notice that since by assumption the set of reactions $\mathcal {R}_a$ satisfy (3.11), then we can repeat step 1 in the proof of Theorem 6.3 to obtain an admissible completion $(\Omega _q, \mathcal {R}_q )$ whose reactions satisfy (3.11). Moreover, since we assume that for every $R \in \mathcal {R}_a$ we have that $\pi _A R =0$ we can apply step 2 in the proof of Theorem 6.3 in order to obtain an admissible completion that satisfies (3.11) and that is conservative.

Finally since the reactions in the set $$\mathcal {R}_a $$ are not in the cycles of the kinetic system $$(\Omega , \mathcal {R}, \mathcal {K} ) $$ we can argue as in step 3 in the proof of Theorem 6.3 to obtain the desired conclusion. Indeed, by construction the vector $$\zeta ^T \in \mathbb {R}^{|\mathcal {R}|}$$ in that proof is such that $$\zeta (i)=0$$ for every *i* such that $$R_i \in \mathcal {R}_a$$. The same holds for the vectors $$W_1 $$ and $$W_2 $$ constructed in that proof. $$\square $$

In the following proposition we prove that if $(\Omega , \mathcal {R}, \mathcal {K} )$ is a kinetic system that does not satisfy the detailed balance property and one of the cycles in the kinetic system contains only reactions that are constrained, then the kinetic system does not admit a closed admissible completion.

#### Proposition 6.8

Assume that the bidirectional kinetic system $(\Omega , \mathcal {R}, \mathcal {K} )$ is such that there exists a cycle $c \in \mathcal {C}$ with

6.1 $$\begin{aligned} \sum _{j=1}^{|\mathcal {R}|/2} \mathcal {E} (R_j) c(j) \ne 0. \end{aligned}$$

Assume moreover that every $R \in c$ is such that $R \in \mathcal {R}_a$ where $\mathcal {R}_a$ is the set of constrained reactions. Then the kinetic system $(\Omega , \mathcal {R}, \mathcal {K} )$ does not admit a closed completion.

#### Proof

Assume that the chemical network $(\Omega _c, \mathcal {R}_c)$ is an admissible completion of $(\Omega , \mathcal {K} )$. We now prove that $c \in \mathcal {C}_c$. Indeed since the completion is admissible we have that given a reaction $R \in c$ it holds that there exists a unique completion $\overline{R}$ of *R* in $\mathcal {R}_c$ and this completion is such that $\pi _{\Omega _c \setminus \Omega } \overline{R} =0$. As a consequence we have that the fact that $\sum _{j=1}^{|\mathcal {R}|} c(j) R_j=0$ implies that $\sum _{j=1 }^{|\mathcal {R}_c|} c(j) \overline{R}_j=0$. Therefore $c \in \mathcal {C}_c$. It follows that for every choice of concentrations $n_{\Omega _c \setminus \Omega }$, the corresponding rate function $\mathcal {K}_c$ defined as

$$ \mathcal {K}_c(R)=\mathcal {K}(R) \prod _{i \in \Omega _c \setminus \Omega } n_i^{R(i)} \ \forall R \in \mathcal {R}_a $$

is such that

$$\begin{aligned}&\sum _{j=1}^{|\mathcal {R}_c|/2} \mathcal {E} (R_j) c(j)= \sum _{j=1}^{|\mathcal {R}_c|/2} \mathcal {E} ( \pi _\Omega R_j) c(j) + \sum _{j=1}^{|\mathcal {R}_c|/2} c(j) \sum _{i \in \Omega _c \setminus \Omega } R_j(i) \log (n_i) \\&\quad = \sum _{j=1}^{|\mathcal {R}_c|/2} \mathcal {E} ( \pi _\Omega R_j) c(j) \ne 0. \end{aligned}$$

In the above computation we have that $\mathcal {R}_c = \{ R_j\}_j$. This implies that we have that there is no completion of $(\Omega , \mathcal {R}, \mathcal {K})$ satisfying the detailed balance property. $\square$

Notice that in the above proposition, we need all the reactions in a cycle to be constrained reactions in order to have the kinetic system lacking a closed admissible completion. If instead only few reactions on the cycles are constrained it might still be possible to obtain a closed completion as shown in the following example.

#### Example 6.9

Consider the kinetic system $(\Omega , \mathcal {R}, \mathcal {K} )$ where $\Omega := \{ 1,2,3,4\}$ and where the reactions are

$$ (1) \leftrightarrows (2) ,\quad (2) \leftrightarrows (3) , \quad (3) \leftrightarrows (4), \quad (4) \leftrightarrows (1), $$

i.e.

$$ {\textbf {R}} =\left( \begin{array}{cccc} -1 & 0 & 0 & 1 \\ 1 & -1 & 0 & 0 \\ 0 & 1 & -1 & 0 \\ 0 & 0 & 1 & -1 \\ \end{array}\right) . $$

Assume that the rate function $\mathcal {K}$ is such that the kinetic system does not have the detailed balance property. We assume moreover that the reaction $(2) \leftrightarrows (3)$ , i.e. the reaction $(0,-1,1,0)^T$ is constrained. We consider the following admissible completion $\Omega _c:= \{ 1,2,3,4, 5, 6\}$ and the following matrix of the reactions

$$ {\textbf {R}}_c =\left( \begin{array}{cccc} -1 & 0 & 0 & 1 \\ 1 & -1 & 0 & 0 \\ 0 & 1 & -1 & 0 \\ 0 & 0 & 1 & -1 \\ 0 & 0 & 0 & -1 \\ 0 & 0 & 0 & 1 \\ \end{array}\right) . $$

Notice that the chemical system $(\Omega _c, \mathcal {R}_c)$ does not have cycles, is conservative and all the reactions satisfy (3.11), hence, for every choice of rate function $\mathcal {K}_c$ we have that the kinetic system $(\Omega _c, \mathcal {R}_c, \mathcal {K}_c)$ is closed. Therefore the original system $(\Omega , \mathcal {R}, \mathcal {K} )$ has an admissible closed completion.

## Kinetic Systems with Fluxes

Let $(\Omega , \mathcal {R}, \mathcal {K} )$ be a kinetic system. Assume that *U* and *V* are as in Definition 4.1. We consider now kinetic systems with fluxes of substances in $U \subset \Omega$. The concentration of the substances $i \in U$ are fixed at some given values $n_U=(n_i)_{i \in U}$. In other words we assume that the concentrations of substances in the network evolve according to the following system of ODEs

7.1 $$\begin{aligned} \frac{d n (t) }{dt } = \sum _{ R \in \mathcal {R}_s } R J_R(n) + J^{E} (t),\ n_0 \in \mathbb {R}_*^N \ \pi _U n_0=n_U \end{aligned}$$

where $J^E(t) = \sum _{i \in U} J^E_i (t)$ with $J^E_i(t):= - e_i \sum _{ R \in \mathcal {R}_s } R(i) J_R(n)$. Later we will use the notation $(\Omega , \mathcal {R}, \mathcal {K} )$ to refer to a kinetic system with fluxes in *U*.

We stress that the kinetic system with fluxes in (2.9) does not have the same stoichiometry and in particular does not have the same conservation laws as the kinetic system $(\Omega , \mathcal {R}, \mathcal {K} )$. Indeed, assume that $m \in \mathcal {M}$. Multiplying equation (2.9) by $m^T$ we obtain that

$$ \frac{d m^T n (t) }{dt } = m^TJ^{E}. $$

As a consequence, unless $\pi _U m =0$, we have that *m* is not a conservation law of the kinetic system with fluxes.

### Long-Time Behaviour of Kinetic Systems with Fluxes

In this section we prove that when the kinetic system $$(\Omega , \mathcal {R}, \mathcal {K} ) $$ satisfies the detailed balance property and we consider the kinetic system with fluxes in *U* that take equilibrium values, i.e. $$n_U $$ is given by (5.2) then the free energy *F* defined as in (3.9) is non increasing and the dissipation $$\mathcal {D}= - \partial _t F $$ is zero at the steady states of the system of ODEs (7.1). This allows us to study the long-time behaviour of the solution to (7.1) and to obtain the following result.

#### Theorem 7.1

Assume that the kinetic system $$(\Omega , \mathcal {R}, \mathcal {K} )$$ satisfies the detailed balance property. Let *U* and *V* be as in Definition 4.1. Assume that $$U\subset \Omega $$ and $$n_U$$ are such that the assumptions of Proposition 5.1 hold, hence $$n_U $$ is given by (5.2). Consider the system of ODEs with fluxes (7.1) corresponding to $$(\Omega , \mathcal {R}, \mathcal {K}, n_U )$$. Then we have that

7.2 $$\begin{aligned} \overline{J}^E_i:=\int _0^\infty J_i^E (t) < \infty , \quad \forall i \in U \end{aligned}$$

where *n* is the solution to (7.1). Assume that the system of ODEs with fluxes (7.1) admits only positive steady states. Then, for every $n_0\in \mathbb {R}_*^N$ that is such that $\pi _U n_0 =n_U$ we have that $\lim _{t \rightarrow \infty } n(t)= e^{-E}$ where *E* is the unique energy of $(\Omega , \mathcal {R}, \mathcal {K})$ that is such that

7.3 $$\begin{aligned} m^T e^{- E }- m^T n_0 = m^T \overline{J}, \quad \forall m \in \mathcal {M}. \end{aligned}$$

Here $\overline{J} \in \mathbb {R}_*^{N}$ is the vector $\overline{J}^E(j)=\overline{J}_j$ for every $j \in U$ while $\overline{J} (j)=0$ for every $j \in V$.

#### Proof

Let us consider the function $F: \mathbb {R}_*^N \rightarrow \mathbb {R}$ defined as in (3.9)

$$ F(n)= \sum _{j \in \Omega } n_j \left( \log \left( n_j e^{E(j) }\right) - 1 \right) $$

where *E* is an energy of $(\Omega , \mathcal {R}, \mathcal {K} )$.

Notice that using (7.1) and (3.7) and using the fact that $n_U$ is given by (5.2) we deduce that

$$\begin{aligned} \partial _t F (n)&= \sum _{j \in \Omega } \partial _t n_j \left( \log \left( n_j e^{E(j) }\right) \right) = \sum _{j \in V } \partial _t n_j \left( \log \left( n_j e^{E(j) }\right) \right) =\\&\sum _{j \in V } \sum _{R \in \mathcal {R}_s } R(j) J_R(n) \left( \log \left( n_j e^{E(j) }\right) \right) \\&=\sum _{R \in \mathcal {R}_s } K_R \prod _{i \in I(R)} n_i^{- R(i) } \left( 1- \prod _{j\in \Omega } n_j^{ R(j) } e^{ R(j)E(j) } \right) \log \left( \prod _{j \in V } n_j^{R(j)} e^{R(j) E(j) }\right) \\&= \sum _{R \in \mathcal {R}_s } K_R \prod _{i \in I(R)} n_i^{- R(i) } \left( 1- \prod _{j\in V } n_j^{ R(j) } e^{ R(j)E(j) } \right) \log \left( \prod _{j \in V } n_j^{R(j)} e^{R(j) E(j) }\right) . \end{aligned}$$

As a consequence we have that $$ \partial _t F (n) \le 0$$ for every $$n \in \mathbb {R}_*^N$$ and that $$ \partial _t F (n) = 0$$ if and only if $$n(i)=e^{- E(i) }$$ for every $$ i \in V $$. Notice that this in particular implies that there exists a constant $$c>0 $$ such that

$$ \left| F(n(t)) \right| = \left| \sum _{j \in \Omega } n_j \left( \log \left( n_j e^{E(j) }\right) - 1 \right) \right| \le c, \quad {\forall t \ge 0}. $$

Notice that this implies that there exists a constant *k* such that $\sum _{j \in \Omega } n_j(t) \le k$ for every $t>0$. Hence for every $j \in \Omega$ it holds that $n_j (t) < \infty$ for every $t>0$. On the other hand notice that for every $m \in \mathcal {M}$ we have that

$$ m^T n(t) - m^T n_0 =m(1) \int _0^t J^E(s) ds. $$

From this we obtain (7.2). Taking the limit as $t \rightarrow \infty$ we deduce that

$$ m^T \lim _{ t \rightarrow \infty } n(t) =m^T \overline{J} + m^T n_0. $$

Now we prove that given an $$n_0 \in \mathbb {R}_*^N $$, there exists an unique energy *E* of $$(\Omega , \mathcal {R}, \mathcal {K} ) $$ such that $$m^T e^{-E} =m^T \overline{J} + m^T n_0$$. Indeed assume that there exists two energies $$E_1 $$ and $$E_2$$ that satisfy (7.3). Then since $$E_1, E_2$$ are energies for the same kinetic system $$(\Omega , \mathcal {R}, \mathcal {K} ) $$ we have that $$R^T (E_1- E_2) =0$$. As a consequence we obtain that there exists a conservation law $$\overline{m} \in \mathcal {M} $$ that is such that $$E_1=E_2 + \overline{m} $$. This implies that

$$ 0=\overline{m}^T (e^{-E_1}-e^{-E_2}) = \overline{m}^T (e^{-E_2- \overline{m}}-e^{-E_2}) = \sum _{i \in \Omega } \overline{m}(i) e^{-E_2(i)} (e^{- \overline{m}(i)}-1 ). $$

Notice that $\overline{m}(i) e^{-E_2(i)} (e^{- \overline{m}(i)}-1 ) \ge 0$ for every $i\in \Omega$ and as a consequence we need $\overline{m}(i) =0$ for every $i \in \Omega$ in order to have $0 = \sum _{i \in \Omega } \overline{m}(i) e^{-E_2(i)} (e^{- \overline{m}(i)}-1 )$. Therefore $E_1=E_2$. This implies that for a given initial datum there is a unique energy such that (7.3) holds.

As a consequence the restriction of *F* to the class of solutions satisfying (7.3) is a radially unbounded Lyapunov functional and $$F(n)=0$$ if and only if $$n=e^{-E} $$ for the unique energy of $$(\Omega , \mathcal {R}, \mathcal {K}) $$ that satisfies (7.3). We then deduce the desired conclusion. $$\square $$

When $$|U | =1 $$ we have the following result.

#### Corollary 7.2

Assume that $(\Omega , \mathcal {R}, \mathcal {K} )$ satisfies the detailed balance condition. Consider the corresponding system with fluxes in $1\in \Omega$, i.e. $(V, \mathcal {R}_V, \mathcal {K}, n_1)$ with $V:=\Omega \setminus \{ 1\}$. Assume that one of the two conditions of Corollary 5.2 holds. Then we have that the solution *n* to (7.1) satisfies (7.2). Assume that the system of ODEs with fluxes (7.1) admits only positive steady states. Then, for every $n_0\in \mathbb {R}^N$ such that $\pi _U n_0 =n_U$ we have that $\lim _{t \rightarrow \infty } n(t)= e^{-E}$ where *E* is the unique energy of $(\Omega , \mathcal {R}, \mathcal {K})$ that is such that (7.3) holds.

#### Proof

The proof of this statement follows by the fact that, as explained in the proof of Corollary 5.2, under the assumptions of the corollary for every value of $n_1$ there exists an energy *E* of the kinetic system $(\Omega , \mathcal {R}, \mathcal {K} )$ that is such that $n_1 = e^{- E(1) }$. The statement then follows by Theorem 7.1. $\square$

### Reduced Kinetic Systems and Kinetic Systems with Fluxes

Clearly there is a relation between the kinetic system with fluxes $(\Omega , \mathcal {R}, \mathcal {K}, n_U)$ and the reduced kinetic system $(\Omega , \mathcal {R}, \mathcal {K} [ n_U])$. In this section we clarify how the two systems are related.

#### Lemma 7.3

(Reduced system and kinetic system with fluxes) Let $(\Omega , \mathcal {R}, \mathcal {K} )$ be a kinetic system. Let *U* and *V* be two sets as in Definition 4.1 and let $n_U:= \{ n_i \}_{i \in U }$. Then the function $t \mapsto n(t) \in \mathbb {R}_*^{|V|}$ is the solution to the system of ODEs (2.7) corresponding to the reduced system $(V, \mathcal {R}_V, \mathcal {K} [ n_U ])$ with initial condition $n_0 \in \mathbb {R}_*^{|V|}$ if and only if the function $t \mapsto \overline{n} (t) \in \mathbb {R}_*^N$ defined as $\overline{n} = ( n, n_U )$ is the solution to (7.1) with initial condition $\overline{n}_0 = ( n_0, n_U )$.

#### Proof

Notice that by the definition of reduced system, and in particular of $\mathcal {K} [n_U]$, we have that *n* is such that

$$\begin{aligned} \frac{ d n }{dt }&= \sum _{R \in (\mathcal {R}_V)_s } R K_R[n_U] \prod _{i \in I(R)} n_i^{- R(i) } \\&= \sum _{R \in (\mathcal {R}_V)_s} R \prod _{i \in I(R)} n_i^{- R(i) } \sum _{\overline{R} \in \pi _V^{-1} (R) } K_{\overline{R} } \prod _{i \in I(\overline{R} ) \cap U } n_i^{- \overline{R}(i) } \\&= \sum _{R \in (\mathcal {R}_V)_s } \sum _{\overline{R} \in \pi _V^{-1} (R) } R \prod _{i \in I( \overline{R})\cap V } n_i^{- \overline{R}(i) } K_{\overline{R} } \prod _{i \in I(\overline{R} ) \cap U } n_i^{- \overline{R}(i) } \\&= \sum _{R \in (\mathcal {R}_V)_s} \sum _{\overline{R} \in \pi _V^{-1} (R) } R K_{\overline{R} } \prod _{i \in I( \overline{R}) } \overline{n}_i^{- \overline{R}(i) } \\&= \sum _{\overline{R} \in \mathcal {R}_s} ( \pi _V \overline{R}) K_{\overline{R} } \prod _{i \in I( \overline{R}) } \overline{n}_i^{- \overline{R}(i) } =\pi _V \left( \sum _{\overline{R} \in \mathcal {R}_s} \overline{R} K_{\overline{R} } \prod _{i \in I( \overline{R}) } \overline{n}_i^{- \overline{R}(i) }\right) =\pi _V \left( \frac{ d \overline{n} }{dt } \right) . \end{aligned}$$

Since by the definition of the fluxes $$J^E $$ in (7.1) we have that $$\pi _U\left( \frac{ d \overline{n} }{dt }\right) =0 $$ the desired conclusion follows. $$\square $$

Notice that when the assumptions of Proposition 5.1 and Corollary 5.2 are satisfied we have that if the kinetic system $$(\Omega , \mathcal {R}, \mathcal {K} ) $$ satisfies the detailed balance condition, then the reduced kinetic system $$(V, \mathcal {R}_V, \mathcal {K} [n_U] ) $$ satisfies the detailed balance condition. As a consequence of Proposition 3.8, the system of ODEs (2.7) corresponding to $$(V, \mathcal {R}_V, \mathcal {K} [n_U] ) $$ has a unique steady state for each compatibility class and the steady state is globally stable. Since, as proven in Lemma 7.3 the dynamics of the system of ODEs (2.7) corresponding to $$(V, \mathcal {R}_V, \mathcal {K} [n_U] ) $$ is essentially the same as the one of the kinetic system with fluxes $$(\Omega , \mathcal {R}_, \mathcal {K}, n_U ) $$ described by the ODEs (7.1) when the initial datum in *U* is given by $$n_U $$, this provides an alternative way of proving Theorem 7.1.

We conclude this section by proving that that the change of free energy in a kinetic system with fluxes is due to the contribution of an external source of energy and due to the dissipation of energy in the reduced kinetic system $(U, \mathcal {R}_V, \mathcal {K} [n_U])$.

#### Proposition 7.4

Assume that the kinetic system $(\Omega , \mathcal {R}, \mathcal {K} )$ satisfies the detailed balance property. Let *U* and *V* be as in Definition 4.1. Assume that $n_U \in \mathbb {R}_*^{|U|}$. Consider the reduced system $(V, \mathcal {R}_V, \mathcal {K}[ n_U ])$. Assume that every $R \in \mathcal {R}$ is not a reaction with a zero-*V*-reduction and that every $R \in \mathcal {R}_V$ is a 1-1 reaction. Assume that *n* is the solution to the system of ODEs with fluxes (7.1) corresponding to $(\Omega , \mathcal {R}, \mathcal {K}, n_U )$. Then

$$ \partial _t F (n)= - \mathcal {D}_R(n) +J^{ext}(n). $$

where *F* is given by (3.9) for an energy *E* of $(\Omega , \mathcal {R}, \mathcal {K} )$ and

$$\begin{aligned} &  \mathcal {D}_R(n)\\ &  \quad = \sum _{R \in \mathcal {R}_s } K_R[n_U] \prod _{i \in I(R) \cap V} n_i^{- R(i) } \left( \frac{ K_{-R}[n_U] }{K_{R}[n_U]} \prod _{i \in V} n_i^{ R(i) }-1\right) \log \left( \frac{K_{-R}[n_U] }{K_R [n_U] } \prod _{j \in V } n_j^{R(j)}\right) , \quad n \in \mathbb {R}_+^N \end{aligned}$$

while

$$ J^{ext}(n):= \sum _{j \in U} J^E_j(t) \log (n_j e^{E(j)}). $$

#### Proof

By the definition of *F* we have that

$$\begin{aligned} \partial _t F&= \sum _{j \in \Omega } \partial _t n_j \left( \log \left( n_j e^{E(j) }\right) \right) = \sum _{j \in V } \sum _{R \in \mathcal {R}_s } J_R(n) \left( \log \left( n_j^{R(j) } e^{E(j) R(j) }\right) \right) \\&+ \sum _{j \in U } \sum _{R \in \mathcal {R}_s } J_R(n) \left( \log \left( n_j^{R(j) } e^{E(j) R(j) }\right) \right) + \sum _{j \in U } J^E_i(t) \left( \log \left( n_j e^{E(j) }\right) \right) \\&=\sum _{R \in \mathcal {R}_s } K_R \prod _{i \in I(R)} n_i^{- R(i) } \left( 1- \prod _{j\in \Omega } n_j^{ R(j) } e^{ R(j)E(j) } \right) \log \left( \prod _{j \in \Omega } n_j^{R(j)} e^{R(j) E(j) }\right) \\&+ J^{ext}(n) \end{aligned}$$

Now notice using the relation between the rates $$K_R $$ and $$K_R[n_U]$$ we deduce that

$$\begin{aligned} \mathcal {D}[n]&= \sum _{R \in \mathcal {R}_s } K_R \prod _{i \in I(R)} n_i^{- R(i) } \left( 1- \prod _{j\in \Omega } n_j^{ R(j) } e^{ R(j)E(j) } \right) \log \left( \prod _{j \in \Omega } n_j^{R(j)} e^{R(j) E(j) }\right) \\&= \sum _{R \in \mathcal {R}_s } K_R[n_U] \prod _{i \in I(R) \cap V} n_i^{- R(i) } \left( 1- \prod _{j\in \Omega } n_j^{ R(j) } e^{ R(j)E(j) } \right) \log \left( \prod _{j \in \Omega } n_j^{R(j)} e^{R(j) E(j) }\right) \\&= \sum _{R \in \mathcal {R}_s } \left( K_R[n_U] \prod _{i \in I(R) \cap V} n_i^{- R(i) }- K_{-R}[n_U] \prod _{i \in F(R) \cap V} n_i^{ R(i) }\right) \log \left( \prod _{j \in \Omega } n_j^{R(j)} e^{R(j) E(j) }\right) . \end{aligned}$$

where in the last equality above we have used the fact that the detailed balance property of $(\Omega , \mathcal {R}, \mathcal {K} )$ implies that

$$ K_R[n_U] \prod _{i \in I(R) \cap V} n_i^{- R(i) } \prod _{j\in \Omega } n_j^{ R(j) } e^{ R(j)E(j) } = K_{-R}[n_U] \prod _{i \in F(R) \cap V} n_i^{ R(i) }. $$

Similarly we have that

$$ \prod _{j \in \Omega } n_j^{R(j)} e^{R(j) E(j) } = \prod _{j \in \Omega } n_j^{R(j)} \frac{K_{-R}}{K_R } = \prod _{j \in V } n_j^{R(j)} \frac{K_{-R}[n_U] }{K_R [n_U] }. $$

As a consequence

$$\begin{aligned}&\mathcal {D} [n] = \sum _{R \in \mathcal {R}_s } \left( K_R[n_U] \prod _{i \in I(R) \cap V} n_i^{- R(i) }- K_{-R}[n_U] \prod _{i \in F(R) \cap V} n_i^{ R(i) }\right) \\ &\log \left( \frac{K_{-R}[n_U] }{K_R [n_U] } \prod _{j \in V } n_j^{R(j)}\right) \\&\quad = \sum _{R \in \mathcal {R}_s } K_R[n_U] \prod _{i \in I(R) \cap V} n_i^{- R(i) } \left( 1- \frac{ K_{-R}[n_U] }{K_{R}[n_U]} \prod _{i \in V} n_i^{ R(i) }\right) \\&\log \left( \frac{K_{-R}[n_U] }{K_R [n_U] } \prod _{j \in V } n_j^{R(j)}\right) \\&\quad =\mathcal {D}_R[n]. \end{aligned}$$

$$\square $$

#### Remark 7.5

Notice that *F* is the free energy of the kinetic system with fluxes $(\Omega , \mathcal {R}, \mathcal {K}, n_U )$. We obtain that

$$ \partial _t F =- \mathcal {D}_R + J^{ext}. $$

Here $$ \mathcal {D}_R $$ is the dissipation of free energy and $$J^{ext} (n) $$ is the external source of free energy due to the in and out fluxes. Notice that $$J^{ext}=0$$ only if $$\pi _U n = e^{- E} $$. Hence the source of free energy due to external fluxes is equal to zero only if the concentration of substances in the set *U* are chosen at equilibrium values. Moreover, notice that $$\mathcal {D}_R[n] =- \partial _t F_V $$ where $$F_V $$ is the free energy of the reduced kinetic system $$(\Omega , \mathcal {R}, \mathcal {K} [n_U]) $$, i.e.

$$ F_V [\pi _V n ] := \sum _{i \in V} n_i \left( \log (n_i e^{E_V(i) } )-1 \right) , $$

where $$E_V $$ is an energy of the reduced kinetic system $$(\Omega , \mathcal {R}, \mathcal {K} [n_U]) $$. Notice that if $$(\Omega , \mathcal {R}, \mathcal {K} [n_U]) $$ satisfies the detailed balance property, then $$\mathcal {D}_R[n] \le 0 $$, where $$n \in \mathbb {R}_+^N $$. While if $$(\Omega , \mathcal {R}, \mathcal {K} [n_U]) $$ does not satisfy the detailed balance property, then there exists a constant $$c>0 $$ such that $$\mathcal {D}_R[n] \le - c$$ for $$n \in \mathbb {R}_+^N $$. In this sense we can say that considering the completion $$(\Omega , \mathcal {K}, \mathcal {R}) $$ allows to measure the lack of equilibrium of the reduced kinetic system $$(V, \mathcal {R}_V, \mathcal {K} [n_U])$$.

## Conclusions

As already mentioned in the introduction, every isothermal kinetic system which does not exchange substances with the environment must satisfy the detailed balance property in order to be thermodynamically consistent (see Falasco and Esposito (2025)). However many biochemical reaction networks are modelled via effective systems that do not satisfy the property of detailed balance. A relevant question is whether these systems can be obtained as approximations of thermodynamically consistent systems. The most important result that we prove in this paper is that every open kinetic system can be obtained by freezing the concentrations of some substances in a larger kinetic system that is closed.

We stress that the completed kinetic system that we construct might have many more substances than the original open kinetic system. However the argument that we use can be optimized in order to reduce the number of substances that we need to add in order to construct a closed kinetic system. A relevant problem is to determine which open kinetic systems can be obtained as the reduction of a closed kinetic system in which only few concentrations can be frozen.

We stress that freezing the concentrations of certain chemicals at constant values is not the only way in which we can obtain non-equilibrium effects. We can imagine an exchange of matter taking place at the same time scale as the evolution inside the kinetic system, for instance we might have that the concentration $$n_F $$ of a certain substance *F* is given by

8.1 $$\begin{aligned} \frac{d}{ dt } n_F =J_F + \alpha (n_{ext}- n_F ). \end{aligned}$$

Here $$\alpha >0 $$ and $$J_F$$ represent the changes in the concentration of $$n_F $$ due to the dynamics taking place in the kinetic system under consideration. Instead, $$\alpha n_{ext}$$ represents the influx of substance coming from the environment, while the term $$-\alpha n_F$$ accounts for the outflux of substance from the kinetic system to the environment. In that case the concentration of the substance *F* is not frozen, but it changes in time. Examples of models in which the concentration of a substance or of many substances change in time due to outfluxes and influxes of matter are for instance models in which there is an active or passive transfer of chemicals through a membrane.

Finally let us mention that the problem of determining if a biological systems can be obtained starting from a larger system for which the detailed balance property holds is relevant also for non-spatial homogeneous systems, modelled by PDEs. It would be interesting to study if a theorem analogous to the completion theorem, Theorem 6.3, holds for certain classes of PDEs, for instance for advection-diffusion equations.
